# Provincial Dietary Intake Study (PDIS): Energy and Macronutrient Intakes of Children in a Representative/Random Sample of 1–<10-Year-Old Children in Two Economically Active and Urbanized Provinces in South Africa

**DOI:** 10.3390/ijerph17051717

**Published:** 2020-03-05

**Authors:** Nelia P. Steyn, Johanna H. Nel, Sonia Malczyk, Linda Drummond, Marjanne Senekal

**Affiliations:** 1 Division Human Nutrition, University of Cape Town, UCT Medical campus, Anzio Road, Anatomy Building, Observatory 7925, Cape Town, South Africa; soniamalczyk@gmail.com (S.M.); linda@linda-drummond.com (L.D.); Marjanne.senekal@uct.ac.za (M.S.); 2 Department of Logistics, Stellenbosch University, 7600 Stellenbosch, South Africa; jhnel@sun.ac.za

**Keywords:** dietary intakes, energy, macronutrients, children 1–<10-years-old, nutrition, double burden of malnutrition

## Abstract

The double burden of malnutrition is still prevalent in South Africa, hence the importance of a dietary survey to identify risks of under- and over-nutrition. A multistage stratified cluster random sampling design was applied in two economically active provinces, Gauteng (GTG) (N = 733) and Western Cape (WC) (N = 593). Field workers completed questionnaires, and a 24 h recall with children taking part aged 1–<10-years (N = 1326). Important findings were that 71% and 74%, respectively, of 3–<6-year-olds and 6–<10-year-olds had an energy intake below the estimated energy requirement (EER), while 66% 1–<3-year-olds had intakes above the EER. The percentage of children with a total fat intake below recommended levels decreased as age increased ((51%, 40% and 5%) respectively, for the three age groups). Similarly, the percentage of those who had a total fat intake above the recommendation increased with increasing age (4%, 11% and 26%, respectively, for the three age groups). Saturated fat intake above 10%E was highest in the youngest and oldest children (33% and 32%, respectively). The percentage of children with a free sugars intake above 10%E was 47%, 48% and 52% respectively, and 98–99% had a fibre intake that was less than recommended. Overall, the diet was not healthy, with the main food items being very refined, and the diet being high in salty snacks and sugary items, and low in fruit, vegetables and legumes.

## 1. Introduction

The growing challenges faced by many low- and middle-income countries are the fact that undernutrition, particularly stunting and micronutrient deficiencies, are still prevalent, while at the same time there is a growing problem of overweight and obesity, both in adults and children [1,2,3,4,5].

De Onis and Mendez [6] have indicated that stunting, which is the most prevalent form of malnutrition, is the best indicator of a child’s wellbeing. It has been shown that growth faltering, which frequently starts in utero and continues into the first two years of life, is associated with many pathological disorders including reduced neurodevelopment and adverse cognitive development. Stunted children may become obese adults, who in turn are more at risk of developing non-communicable diseases (NCDs) [6,7]. Rapid weight gain in infancy is also associated with long-term risk of adult weight gain and development of NCDs [8].

In a recent publication of the same children whose dietary results are studied here, nutritional status by means of anthropometry was determined [9]. Stunting was found to be 39% and 23% in 1- and 2-year-olds, respectively. Overweight and obesity were found to be 23% and 14% in 1-year-olds, and 11% and 9% in 2-year-olds, and those who were both stunted and overweight were 19% and 6%, respectively, in 1- and 2-year-old children. The double burden of malnutrition is clearly illustrated.

The double burden of malnutrition is commonly found in many low- and middle-income countries [10] and much of this can be largely attributed to the nutrition transition taking place, in conjunction with decreased levels of physical activity [10]. The nutrition transition implies moving from a traditional diet which is high in carbohydrate and fibre and low in sugar and fat to a more contemporary Western diet which is high in fats, saturated fats, sodium and sugar [11]. One of the main drivers of this transition is urbanization [10,12].

A recent review of dietary studies in developing countries by Ochola and Masibo showed what the typical dietary scenario in developing countries is [13]. This review included 50 studies from 42 countries in children and adolescents. The findings were an overall low energy intake, lack of dietary diversity, low intake of fruit and vegetables and micronutrient deficiencies. At the same time the emerging nutrition transition is stressed. This includes an increase of high energy snacks and sweetened beverages, particularly in urban areas.

A similar scenario is painted in West Africa [14]. It was noted that in large cities, children consume energy-dense foods such as sweetened beverages, candies and ice cream. In fact, sweetened beverages were found to be consumed up to seven times as frequently as fruit and vegetables. Overall, there appeared to be strong evidence for an increased intake of dietary energy, fats, sugar, salt, and decreased fruit and vegetable intake.

In South Africa few data are available regarding the diet of children and how the nutrition transition has affected their dietary intake. The only national survey in children was undertaken in 1999, the National Food Consumption Survey (NFCS), with no follow up for comparison to show trends and changes in diet [15]. Evidence from numerous local studies in South Africa with children and adolescents in both urban and rural areas suggests increases in fat, sugar and salt, due to consumption of energy dense snacks, fast food consumption, and sweetened beverages [16,17,18,19]. The Provincial Dietary Intake Study (PDIS) is a follow-up of the NFCS study in two rapidly urbanizing provinces. The aim was to determine the energy and macronutrient intakes of children aged 1–<10-years, food contributors to macronutrient intake, most commonly eaten items, and to determine sociodemographic predictors of energy and selected macronutrient intakes.

## 2. Materials and Methods

### 2.1. Study Area

The two provinces selected were Gauteng (GTG) and the Western Cape (WC), because they are the most rapidly urbanizing and wealthiest provinces, with extensive migration from rural areas to cities in search of jobs and a better quality of life [20].

### 2.2. Structure of the Sample and the Sampling Procedure

Six strata were identified during the design phase, namely two provinces (GTG and WC), with each having three areas of residence: urban formal, urban informal and rural areas. Formal areas include planned developments with roads, infrastructure and brick houses. Informal areas are unplanned developments with housing not made from formal building materials, also referred to as shacks. A rural area is any area that is not classified as urban, and may comprise a tribal area, commercial farm or an informal settlement, and is so designated by Statistics South Africa [21]. All the enumerator areas (EAs) were identified in each stratum. A stratified two-stage sample design was used, with a probability proportional to size sampling of EAs at the first stage, and the systematic sampling of households (HHs) within the EAs at the second stage [22].

The number of HHS per sampling stratum (province and residential area), taking non-response into account was calculated to be N (=175); where the design effect (Deft = 1.3), the estimated proportion of children classified as stunted (*p* = 0.21), and the desired relative standard error (*a* = 0.2) are based on estimations from previous surveys. The p value for stunting was used, since the current study formed part of a larger study, which also looked at anthropometric status [9]. The individual response rate (R_1_ = 0.96) was expected to be higher than the expected household gross response rate (R_2_ = 0.89). The number of eligible individuals per household (d = 1.06) was calculated as the average number of children aged 1–<10- years per household. It was proposed to survey 175 × 6 strata, or 1050 HHs.

For the precision of estimates to be acceptable across regions, experience shows that a minimum of 50 interviews per stratum are needed, so that reliable estimations for indicators under investigation can be obtained. The final sample allocation reflects a power allocation of 0.5, which is between the proportional allocation and the equal size allocation, so that the survey precision in the urban formal areas is comparable with the urban informal and rural areas, with urban informal and rural areas slightly over-sampled. Since the sample sizes of GTG rural, WC rural and urban informal were less than 150, we increased sampling accordingly, to ensure sufficient observations per cell in each age group, with the proposed sample size then being 1050 + 218 = 1268. A total of 84 EAs were selected from the six strata, 25 formal residential, 10 informal residential and 11 rural EAs in GTG, and 18 formal residential, 10 informal residential and 10 rural EAs in the WC.

### 2.3. Selection of Households

Maps of relevant primary sampling units were generated and passed on to the respective fieldwork teams. An estimate was made of the total number of HHs in each EA, to determine the approximate number of qualifying HHs with children within the prescribed age interval in the EA. A listing of eligible HHs was compiled in all selected EAs, which served as a sampling frame for the selection of HHs. HHs (a maximum of 16) were then selected based on a predetermined fixed interval (calculated to be specific to each EA), starting from a randomly determined point. A backup sampling frame was constructed in each EA, by asking members of the 16 selected HHs to identify nearby HHs with women and children of the appropriate age.

### 2.4. Selection of Children within Households

One child in each randomly selected HH was included in the survey. If there was more than one child present in the prescribed age interval within a HH, then all eligible children in the HH in age order were numbered for random selection of one child, using a “Random Number Table” designed for this purpose.

The inclusion criteria for the current study were as follows: children aged 1–<10-years (12–119 months) old; male or female; availability of a parent/primary caregiver to provide consent; and availability of a parent/primary caregiver to assist with completion of the research questionnaires. The exclusion criteria were as follows: children who were mentally or physically handicapped; children who were on a prescribed diet, e.g., for Type 1 diabetes; children who were ill at the time of the visit or were ill during the past 24 h; children whose mothers/caregivers were unable to respond to, or appeared to be incapable of responding or providing reliable information; children whose mother/caregiver was under the influence of alcohol/drugs or was under 15-years-old.

Sampling weights were calculated to adjust for the oversampling in the rural and urban informal areas and the number of children in the 1–<3, 3–<6, and 6–<10-year age groups, bearing in mind the survey design. The final weight was the product of the proportional and realization weights. The final post-hoc stratification weighting reflects the census population of the Western Cape and Gauteng provinces.

### 2.5. Fieldwork Teams

Each province was led by a provincial dietitian, who was responsible for the overall management of the research teams in the two provinces. Both GTG and the WC had two research teams each: teams included a team leader and two pairs of field workers for a total of 11 team members per province. The field workers were selected based on a minimum level of grade 12, i.e., completion of high school, as well as other experience in surveys and in field work. Before data collection began, team leaders and field workers received a week-long extensive training session, according to a manual which had been developed for the purpose of the study, facilitated by experienced researchers in anthropometric measurements, as well as the delivery of sociodemographic questionnaires and other questionnaires. After each training module the field workers practiced using the questionnaires through role play sessions with each other. The training session included standardizing the anthropometric measurements done by the field workers against a trained and experienced anthropometric researcher. At the end of the week, the field workers did a practical and written test based on case studies. Field workers who did not achieve a certain percentage were not selected. Field workers carried their manuals in the field during the period of the study.

### 2.6. Measures

#### 2.6.1. Sociodemographic Questionnaire

The questionnaire comprised questions about the child: birth date, gender, birth order, schooling/day centre, and dietary supplements from clinics. Questions about the family and household: head of household, primary caregiver, marital status of mother, education and employment status of mother and father, type of house, availability of electricity or other energy devices, drinking water, type of toilet, and household density. These variables were selected as they were used in the NFCS and many were found to be significant predictors of nutritional status [23].

A wealth index was calculated as indicated by the World Bank [24] and applied in the 2016 South African Demographic and Health Survey [25]. Principal component analysis was used to estimate relative wealth, and this estimation is based on the first principal component. This component contributes to a wealth index that assigns a larger weight to assets that vary the most across HHs, so that an asset found in all HHs is given a weight of zero. The wealth index was based on amenities available in the home and environment and was developed using an iterated principal factor analysis (Supplementary Table S1).

#### 2.6.2. Hunger Scale Questionnaire

Hunger (food security) was measured using the Community Childhood Hunger Identification Project (CCHIP) questionnaire [26], included as Supplementary Table S2. This questionnaire measures household, child and individual level food security. Altogether, there are eight questions in the scale. If any of these are affirmative (answered yes), then a score of one is given. The same scoring system was used in the NFCS in 1999 [15]. A total score of 5–8 indicates that a food shortage is present in the house. A score of 1–4 indicates that the household is at risk of hunger (poor food security) and a score of zero indicates that the house is food secure. These scores were used to calculate an association with selected dietary variables.

#### 2.6.3. Dietary Intake

A 24 hour recall (24 h recall) was done with each participant to determine total energy and nutrient intake. This was done because the literature indicates that the accuracy of reporting own dietary intake in younger children is not good,, but improves between the ages of 8 and 12 years [27]. Consequently, in this study all dietary interviews took place in the presence and with the input of the mother/primary caregiver. For 1–<6-year-old children the mother/caregiver reported on the intake of the child on the previous day, with no input from the child. For 6–<10-year-old children the mother/caregiver and child were interviewed together, to record the dietary intake during the prior 24 h. If the child had been at a day care centre the previous day, they were visited by the fieldworker and meals and portion sizes were determined for the 24 h in question. All weekdays and Sundays were covered proportionally by each team to ensure that potential variation because of day of the week was captured.

A single 24 h recall has been used as the primary instrument for measuring dietary intake in numerous large dietary studies. A common concern with a single 24 h recall is the day-to-day variation in the diet of free-living populations. The magnitude of the mostly random within-person variance varies by nutrient and is largely dependent on cultural and ecological factors. Methodological challenges in estimation of dietary intake may also contribute to within-person error. These errors result in large standard deviations (SDs) in population groups and insignificant regression coefficients. Another important result of exaggerated variation is that the percentage of subjects below or above specified cut-points will be distorted. The National Cancer Institute (NCI) method [28,29], that was developed to distinguish within-person from between-person variation, account for extreme intakes, including zero intake, and allow for adjustment for covariates and association analyses was applied in this study to estimate the usual dietary intake from repeated 24 h dietary recall assessments. For these purposes, two additional 24 h dietary recalls were completed on a subsample of 148 (2nd recall) and 146 (3rd recall) children in the sample. For logistic reasons, this subsample was recruited from the last five EAs visited in each province. The same houses were revisited, and the same children’s caregivers interviewed. Comparison of sociodemographic variables between those who completed one 24 h recall and those who completed repeated recalls showed only two significant differences. The subgroup had more unmarried mothers than the total group (*p* < 0.001) and more black African children (*p* < 0.001) (Supplementary Table S4). Whether the 24 h recall was less, the same or more than the child’s usual intake was also recorded for the total group, as well as for the two additional recalls completed for the subgroup (Supplementary Table S4).

The multiple pass method of the 24 h recall was used to administer the 24 h recall [30]. Essentially the interviewer first went through the previous day’s intake by recording all the food items and drinks that were consumed between waking up in the morning until going to sleep in the evening (and during the night if applicable). Recall was helped by the interviewer going through the daily activities with the participant and linking them to eating occasions. Next, the interviewer prompted the respondent to identify food and drinks that may have been “forgotten,” such as cold drinks, candies and snacks. Information was then recorded regarding when and where the various food items were consumed. Following this, more detailed information was obtained regarding the preparation of the foods and individual ingredients as relevant. Lastly, portion sizes were recorded as accurately as possible for all foods/drinks consumed. A combination of methods was used to determine mixed dishes. When generic recipes were available in the food composition tables, they were used. When no recipes were available, the ingredients were calculated proportionally and added as individual items from the food composition tables. Field workers were taught how to do this during their training programme.

**Figure 1:**
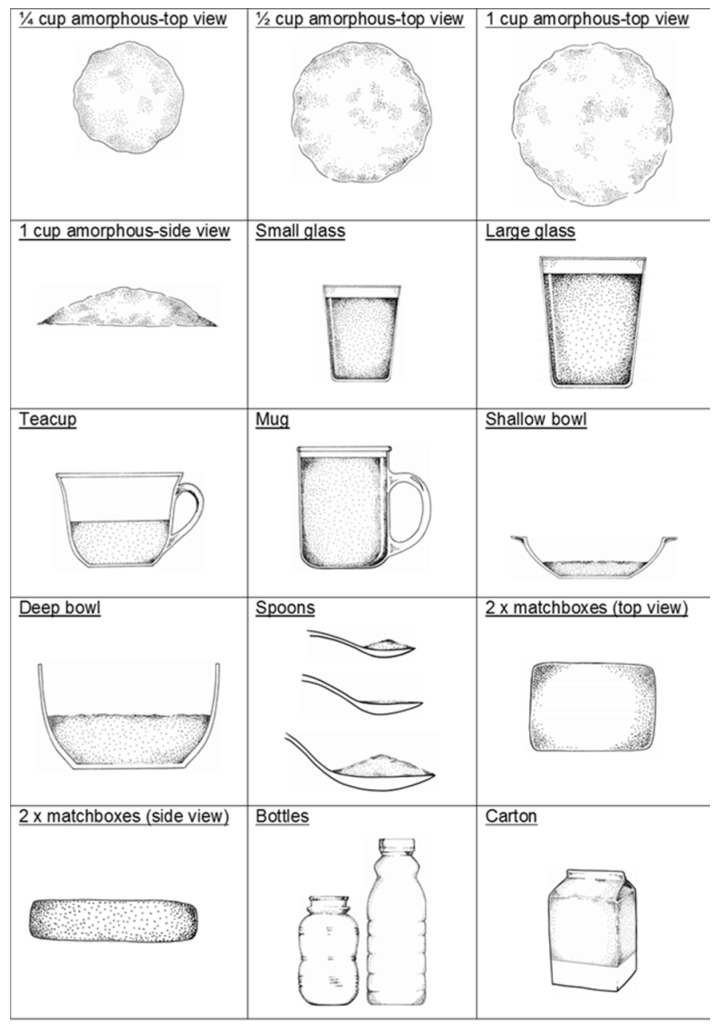
Examples of sketches and measures used in the study.

Portion sizes were obtained using a booklet adapted from the Dietary Assessment and Education Kit (DAEK) [31]. The booklet comprises life size sketches of generic household utensils and crockery (Figure 1) and life size portions of actual foods e.g., different slices of bread varying in size and thickness, to make estimations of portion size as accurately as possible. The sketches have been validated in adolescents [32]. Generic three-dimensional food models made from flour were also used to assist in recording volume measures such as porridge and rice.

Breast milk consumption was quantified by asking mothers whether their child was still receiving breast milk and if yes, the number of feeds the child received during the previous 24 h. Based on the study by Neville et al. [33], we used an estimate of 100mL per feed to calculate the volume of breast milk consumed per day.

#### 2.6.4. Anthropometry of Mothers

The mothers’ height and weight were measured to calculate BMI as weight divided by height squared (kg/m^2^), for inclusion as a sociodemographic indicator in regression analyses with select dietary outcome variables. Electronic digital scales (Scalerite Micro glass bathroom scales, Scalerite, Benrose, GTG, South Africa, capacity 180 kg) and a stadiometer (SECA 213 portable stadiometer, SECA, Hamburg, Germany) were used to determine weight and height, respectively, in accordance with standard procedures [34] by trained field workers. The mothers were weighed in light clothing (without coats, cardigans and shoes) and the reading was recorded to the nearest 100 g.

The heights of mothers were measured without shoes and standing upright on the base board of the stadiometer, with their backs to the vertical rod of the stadiometer, facing the fieldworker. The arms were hung loosely by the side and the head was in the Frankfurt plane. The fieldworker then lowered the headboard until it touched the head (any hairclips/pieces that may have impacted the reading were removed prior to taking the measurement). The reading was taken to the nearest 0.1 cm. Weight and height measurements were repeated and an average was used [34]. BMI was classified as underweight (BMI < 18.5 kg/m^2^), normal weight (18.5 ≤ BMI ≤ 24.9 kg/m^2^), overweight (25.0 ≤ BMI ≤ 29.9 kg/m^2^) or obese (BMI ≥ 30 kg/m^2^) [35].

### 2.7. Data Analyses

After completion of an EA, the questionnaires were checked by the two provincial dietitians, who managed the fieldwork as well as the quality control of data collection in each province. The questionnaires were then dispatched to a central point for data entry. Data analyses were conducted using SAS Version 9.4, SAS for Windows (SAS Institute, Carry, NC, USA). Weighted means, proportions and 95% confidence intervals were calculated by incorporating the complex survey design.

The 24 h recall data were analysed using the South African Food Composition Tables [36]. Trans fat values were analysed using the South African Food Consumption Tables of 2002. Total intakes of energy, macronutrients (carbohydrate, fat and protein) and fibre were calculated.

The prevalence of intakes below the dietary reference intakes (DRIs) [37] for estimated energy requirement (EER), and alignment with the acceptable macronutrient distribution ranges (AMDR) for other macronutrients were also determined. Each child was compared with their own age values within the age groups. Since there is no AMDR for saturated fat or trans fats in children, the latest recommendation by WHO was used, namely intake <10%E and <1%E, respectively [38] (Table 1). In terms of sugar, we used the recommendation of the WHO [39] that intake of free sugars should be less than 10%. “Free sugars include monosaccharides and disaccharides added to foods and beverages by the manufacturer, cook or consumer, and sugars naturally present in honey, syrups, fruit juices, and fruit juice concentrates [36].” Since free sugars are not found in the South African Food Composition Tables, we used the method used to calculate free sugars in New Zealand [40].

Since South Africa has introduced a levy on sugar sweetened beverages, which has resulted in some companies reformulating the amount of sugar added to their products, we checked the relevant beverage labels to ensure that they were in line with the food tables.

Additionally, we identified the percentage of individual food items which contributed at least 4–6% (main 4 items) to energy intake and selected macronutrients, as well as the food items which were most commonly consumed. This was done in the NFCS [15] and helped to provide important information on foods selected to be fortified. We were interested in seeing whether the main food items had changed after nearly two decades and whether there was evidence of the nutrition transition.

The Rao–Scott Chi-square test, considering the complex survey design, was used to test for relationships between province and selected sociodemographic and other characteristics. The independent t-test was used to test for significant differences of usual nutrient intakes between the two provinces. The standard errors used for the independent *t*-tests required additional programming to implement the balanced repeated replication (BRR) when using the NCI method (see Supplementary Table S3 for more detail) [28,29].

A process was followed to select the final multivariate logistic regression model. Variables of importance had been identified, mainly from the 1999 NFCS [15,22]. A bivariate logistic regression analysis, incorporating the complex survey design, was performed to identify the sociodemographic and other factors associated with energy expenditure and other macronutrients.

Variables in the model included: who looks after the child; age and gender of the child; head of the household; marital status of the mother; mother’s employment status; fathers employment status; mother’s education level; father’s education level; BMI of the mother of the child; wealth index quintiles; province; type of residence; ethnicity; and finally, the risk of hunger classification. Significant relationships (*p* < 0.05) were further investigated in multivariate logistic analyses, incorporating the complex sample design. Odds ratio estimates with 95% confidence intervals are reported when using the bivariate and multivariate logistic regressions. The Wald chi-square was used to test the significance of the estimates in the logistic regression. Multicollinearity was addressed by excluding highly correlated variables.

Since no significant differences were found for mean energy and nutrient intakes between areas of residence or gender in both provinces, the data were pooled, and are presented by age group and province.

### 2.8. Ethics

The study was conducted in accordance with the principles of the 2013 Declaration of Helsinki [41], Good Clinical Practice (GCP) and the laws of South Africa. The approval from the Faculty of Health Sciences Human Research Ethics Committee at UCT was obtained on the 18th July (HREC REF:326/2018). Parents or primary caregivers of children provided informed, signed consent. Children aged 6 to 9 years were also asked for verbal assent.

## 3. Results

Table 2 presents general sociodemographic and other characteristics of the sample. The mother looked after the child most of the time as the primary caregiver (70%), followed by a grandparent being the most likely alternative primary caregiver (18%). The latter was significantly more likely in the WC than in GTG. The father was the head of the household in 40% of homes, followed by the grandmother (24%). Overall, 53% of mothers had not completed grade 12, with mothers in WC being significantly more likely to have a grade 12 level of education. Fewer mothers were employed (28%) compared with fathers (65%). Overall, 75% of the sample were black African and 24% were of mixed origin and 88% of the sample lived in urban formal areas. Twenty-five percent of mothers were overweight and 43% were obese, with mothers in the WC being significantly more likely to be obese. Twenty-four percent of HHs were at risk of hunger and 21% had a food shortage in the home.

Children who were 1–<3-years-old had a mean energy intake of 4944 kJ, carbohydrate intake of 163.7 g, protein intake of 34.6 g (17.8 g animal and 12.3 g plant protein) and fibre intake of 10.1 g (Table 3). In terms of AMDR values, their mean percent of energy intake (%E) from carbohydrate was 59%E; protein was 12%E, animal protein was 6%E and plant protein was 4%E. Children who were 3–<6-years-old had a mean energy intake of 5626 kJ, carbohydrate intake of 187.3 g, protein intake of 40.3 g (21.0 g animal and 16.9 g plant protein) and fibre intake of 12.7 g. Their AMDR mean values were 60%E from carbohydrate, 12%E from protein, 6%E from animal protein and 5%E from plant protein. Children who were 6–<10-years-old had a mean energy intake of 6530 kJ, carbohydrate intake of 209 g, protein intake of 46.2 g (24.1 g animal and 20.8 g plant protein) and a fibre intake of 14.1 g. Their AMDR mean values were 57%E from carbohydrate, 12%E from protein, 6%E from animal protein and 5%E from plant protein.

Children who were 3–<6-years-old in GTG had a significantly lower total energy intake than those in WC. (Table 3). Total carbohydrate intake (g) was significantly higher in GTG than in WC in the oldest age group, while the %E from carbohydrate was significantly higher in GTG than in WC in all three age groups. Total protein intake (g) was significantly different between the two provinces in the two younger age groups, and the %E from protein was significantly lower in GTG than in WC in all three age groups. Animal protein intake (g) was lower in GTG than in WC in all three age groups, while %E from animal protein was only significantly lower in GTG than in WC in the two older age groups. Plant protein intake was significantly higher in GTG than in WC for all three age groups, while the %E from plant protein was higher in GTG than in WC in all the age groups. Fibre intake (g) was significantly higher in GTG than WC in the youngest and oldest of the three age groups (Table 3).

In the 1–<3-year-old group, mean fat intake was 39.4 g, saturated fat 12.0 g, cholesterol 106.8 mg, monounsaturated fat 11.4 g, polyunsaturated fat 9.5 g, trans fats 0.8 g, and free sugars 30.9 g (Table 4). Regarding mean % contribution of macronutrients to total energy intake, 30%E came from total fat, 9%E from saturated fat, 9%E from monounsaturated fat, 8%E from polyunsaturated fat, and 10%E from free sugars (Table 4). In the 3–<6-year-old group, mean intake fat intake was 44 g, saturated fat 13.1 g, cholesterol 125.8 mg, monounsaturated fat 13.7 g, polyunsaturated fat 12.2 g, trans fats 0.9 g, and free sugars 33.9 g. Regarding mean % contribution of macronutrients to total energy intake, energy from fat contributed 29%E, saturated fat 9%E, monounsaturated fat 14%E, polyunsaturated fat 8%E and free sugars 10%E (Table 4). In the 6–<10-year-old group, mean fat intake was 56.3 g, saturated fat 15.9 g, cholesterol 153 mg, monounsaturated fat 17.7 g, polyunsaturated fat 17.2 g, trans fat 1.2 g, and free sugars 40.2 g. Regarding AMDR values, energy from fat was 32%E, saturated fat 9%E, monounsaturated fat 10%E, polyunsaturated fat 10%E and free sugars 10%E.

Total fat intake (g) was significantly lower in GTG than in WC in the youngest age group, while %E from fat was significantly lower in GTG than WC in the oldest age group (Table 4). Saturated fat (g) intake was significantly lower in GTG than WC in all three of the age groups, but %E from saturated fat was only significantly lower in GTG than in WC in the two older age groups. Monounsaturated fat intake (g) was significantly lower in GTG than WC in the youngest and oldest age groups, while there was only a difference in the oldest age group for %E from monounsaturated fat. The only significant difference between the two provinces for polyunsaturated fat was for 3–<6-year-old children where those in GTG had a higher %E intake from these fats than those in WC. Trans fat intake was significantly lower in GTG than WC in the two older age groups. Cholesterol intake was significantly lower in GTG than WC, in the youngest and oldest age groups only. There was no difference in free sugars intake (g) or %E from free sugars between the two provinces, for any of the age groups (Table 4).

**Figure 2:**
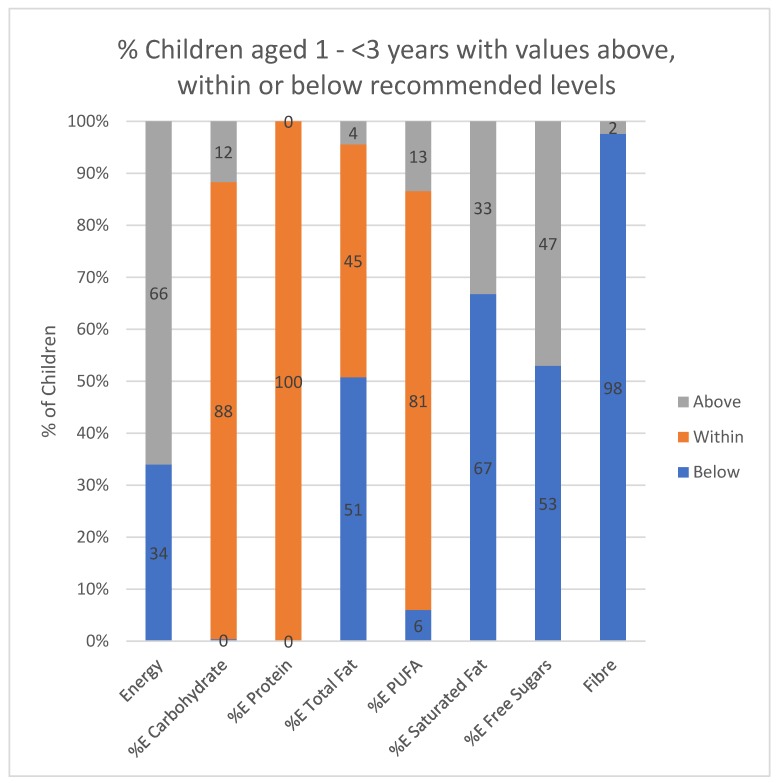

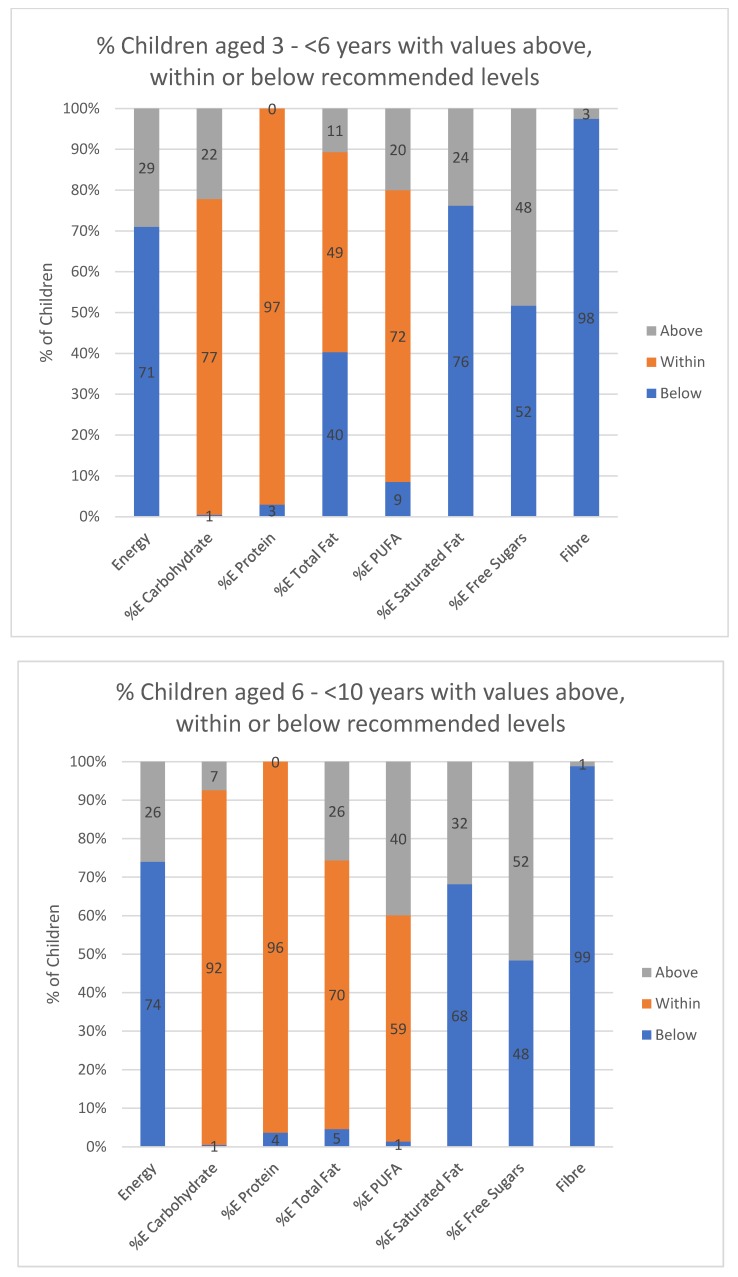
Children aged 1–<3 years; 3–<6 years and 6–<10 years, lying below, within, or above recommended energy and macronutrient intake ranges for their age. EER = Estimated energy requirement (37]: M (1–2 yrs): 4393 kJ; F (1–2 yrs): 4166 kJ; M (3 yrs): 6213 kJ; F (3 yrs): 5837 kJ; (4 yrs): 6552 kJ; F (4 yrs): 6171 kJ; M (5 yrs): 6937 kJ; F (5 yrs): 6514 kJ; M (6 yrs): 7289 kJ; F (6 yrs): 6870 kJ; M (7 yrs): 7699 kJ; F (7 yrs): 7192 kJ; M (8 yrs): 8079 kJ; F (8 yrs): 7573 kJ; M (9 yrs): 8548 kJ; F (9 yrs): 7908 kJ; Acceptable macronutrient distribution range (AMDR): Carbohydrate %E: 45–65%, [37]; %E from protein in 1–3-year-olds: 5–20%, 4–9-year-olds 10–30% [37]. Protein RDA 1–3-year-olds 3 g, 4–8-year-olds 19 g, 9-year-olds 34 g [37]. %E from fat in 1–3-year-olds 30–40%, 4–9-year-olds 25–35% [38], saturated fat is <10%E [38]; Total fibre adequate intake (AI) [37] 1–3-year-olds −13 g, 4–9-year-olds 25 g, and free sugars intake <10%E [38].

Figure 2 depicts the percentage of children in each of the three age groups lying below, within, or above recommended energy and macronutrient levels for their age. More than two thirds of the two older groups (71% and 74%, respectively) had an energy intake below the EER cut-off, while this was only a third (34%) for the 1–<3-year-old group. For all three age groups, carbohydrate intake was within (88%, 77% and 92%, respectively) or above (12%, 22% and 7%, respectively) the AMDR. Protein intake was within the AMDR for all the 1–<3-year-olds, while only 3% and 4%, respectively, of the two older groups had an intake below the recommendation. The percentage of children who had a total fat intake below the AMDR decreased as age increased (51%, 40% and 5%, respectively, for the three age groups). Similarly, the percentage of children who had a total fat intake within and above the recommendation increased with increasing age (4%, 11% and 26%, respectively, for the three age groups). The majority of children in all three age groups had a polyunsaturated fat intake within or above the AMDR. Saturated fat intake greater than 10%E was highest in the youngest and oldest children (33% and 32%, respectively). The percentage of children with a free sugars intake above the recommendation was 47%, 48% and 52%, respectively, in the three age groups. Almost all the children in each of the three age groups had a fibre intake below the recommendation (98%, 98% and 99%, respectively).

The main food contributors to kJ intake in all age groups were maize porridge, salty snacks and potatoes/sweet potatoes (Table 5). Items in the 1–<3-year-old group also included whole milk, while chicken (any type) was a contributor to total energy intake in the 3–<6-year-old group and white bread in the oldest group. Maize porridge and granulated sugar were the main food contributors to carbohydrate intake in all three age groups. In the youngest age group, further contributors to carbohydrate intake were potato/sweet potato and breast milk substitute (BMS); in the middle age group white bread and white rice; and in the oldest age group white bread and sugar-sweetened cold drinks. The main food contributors to protein intake in the two younger age groups were chicken, maize porridge, whole milk and beef (any type). Contributors to fat intake in all groups were salty snacks and chicken. Further contributors in the 1–<3-year-old group were whole milk and BMS and in the two older groups, medium fat margarine and processed meat. Contributors to saturated fat intake were whole milk, salty snacks, processed meats and chicken for the two younger groups, with medium fat margarine replacing chicken in the older group. Main sources of free sugars in all three the age groups included granulated sugar, candies and sweetened cold drinks (carbonated and cordial/squash). The fourth source in the younger age group was yoghurt, while the two older groups had cookies as the fourth source. The main contributors to fibre intake in all groups was maize porridge, with high fibre cereal and potato/sweet potato being sources in the two younger age groups, fresh fruit in the youngest age group only, brown bread in the 3–<6-year-old group and white and brown bread in the 6–<10-year-old group.

The percentage of children who were still being breastfed was 11.7 (n = 39) for 1–<3-year-old and 1.0% (n = 5) for 3–<6-year-old children (Table 6). For children who were breastfed the mean (SD) frequency, the frequency of feeds per day was 3.0 (1.7) for 1–<3-year-olds and 1.8 (0.8) for 3–<6-year-old children, respectively. The mean (SD) breastmilk consumed per day was 300 g (175) (equivalent to 891 kJ) for 1–<3-year-olds and 180 g (84) (equivalent to 534.6 kJ) for 3–<6-year-old children, respectively. The percentage contribution of breastmilk to total energy in those who were breastfed was 18.7% for 1–<3-year-old children and 8% for 3–<6-year-old children.

**Figure 3:**
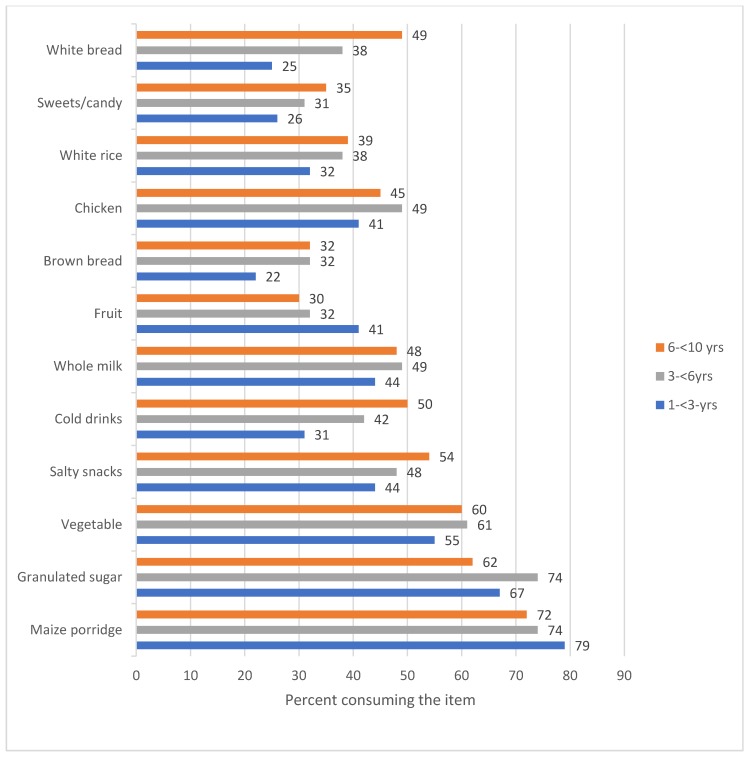
The twelve most commonly consumed food items by all children by age group.

Figure 3 presents the most commonly consumed food items by the three age groups studied. Overall, the main items eaten by all three groups were maize porridge, granulated sugar, a vegetable, salty snacks, whole milk, cold drinks, a fruit, and chicken. However, certain items were more common among certain age groups. In 1–<3-year-old group, maize porridge (79% vs. 74% and 72%) and fruit (41% vs. 30% and 32%) were most commonly consumed. Granulated sugar was most commonly consumed by the 3–<6-year-old group (74% vs. 62% and 67%), and they also commonly consumed vegetables (61% vs. 60% and 55%). The oldest group consumed the most salty snacks (54% vs. 48% and 44%), cold drinks (50% vs. 42% and 31%), white bread (49% vs. 38% and 25%), and candy (35% vs. 31% and 26%).

Table 7, Table 8, Table 9 and Table 10 provide results on bivariate and multivariate logistic regression analysis, with respect to socio-demographic predictors of select variables by age group. For the 1–<3-year-old group, significant predictors (based on multivariate logistic regression analysis) of energy intake below the EER included having an “other “as the head of the household (reference: father), having a mother who completed grade 12 or who had a qualification after grade 12 (reference: did not complete grade 12) were protective factors. Having a mother who completed grade 12 was also a protective factor in the 6–<10-year-old group. In the 3–<6-year-old group, being at risk of hunger or a having a food shortage in the home was also a protective factor (reference: no risk of hunger (Table 7)).

There were no significant predictors of protein intake below the AMDR for the 1–<3-year-old group (Table 8). For 3–<6-year-old children, coming from a household where the mother was the head of household and living in Gauteng were significant predictors of a protein intake below the AMDR. In this same age group, having a mother who has completed grade 12 was protective. In the 6–<10-year-old group, significant predictors of a protein intake below the AMDR included living in GTG (reference: living in WC) and having an overweight or obese mother (reference: underweight/normal weight mother) (Table 8).

There were no significant predictors of total fat intake below the AMDR for 1–<3-year-old children (Table 9). A significant predictor of having a fat intake above the AMDR in the 3–<6-year-old and 6–<10-year-old groups was being of mixed ancestry (reference: black African). A protective factor in the 3–<6-year-old group was having a mother who had completed grade 12. In 6–<10-year-old children, a protective factor was living in an urban informal area (reference: living in a formal urban area) and having a food shortage in the house.

For 1–<3-year-old children, a significant a predictor of intake of free sugars above 10%E was coming from a household where the mother or a grandparent was the head of the household (Table 10). For the 3–<6-year-old group, coming from a household where an “other” was the head of the household was a predictor of a free sugars intake above 10%E, while being at risk of hunger or having a food shortage in the home were protective factors. Predictors of free sugars intake above 10%E in 6–<10-year-old children included having a mother who was employed (reference: not employed), having a father who was employed and being of mixed ancestry.

## 4. Discussion

In the most recent Lancet Series on the double burden of malnutrition, the fact that low- and middle-income countries face a double burden of malnutrition as a result of a global nutrition transition is reiterated [42]. This is also true for the two provinces in South Africa we investigated, as was reported in the paper by Senekal et al. [9]. Popkin et al. [42] mention that a key factor in this transition over and above the transition from more traditional to Western diets is the change in the global food system, that makes less nutritious food cheaper and more accessible. This paper focuses on the energy and macronutrient intake of the 1–<10-year-olds surveyed in the two provinces. Overall, the dietary intake shows elements of both over- and undernutrition in all three of the age groups investigated.

The 1–<3-year-old group have some special nutrition requirements for this period, where some are still receiving breastmilk, or a BMS, and transition to the adult diet is taking place. Children in this age group are mainly still found in the home and are fed by a primary caregiver. According to Mameli et al. [43] several risk factors identified as possible determinants of later-life obesity act within the first 1000 days of life. They identified three different stages in the origin of childhood obesity, namely the prenatal period, breast versus formula feeding and thirdly the complementary diet.

The nutritional benefits of breastmilk versus formula in terms of the prevention of obesity is well accepted [44]. The most widely accepted explanation for this is that breastfed infants have a lower growth velocity than formula-fed infants, which is thought to be a protective factor for later life obesity [44]. The fast growth curve of formula-fed infants may be the result of a higher plasma Insulin-like Growth Factor (IGF)-1, which may be a potential consequence of endocrine modulation [45]. In South Africa, exclusive breastfeeding decreases with age from 44% at 0–1 month to 24% at 4–5 months and 45% are fed using a bottle with a nipple [25]. Furthermore, while it is recommended to breastfeed until 24 months [46] in the current study only 12% of children in the youngest group still received breastmilk. In the first 1000 days, early solid feeding has been shown to be associated with formula-feeding in infants who received solid foods before four months of age [47]. These infants had a six-fold increase in the odds of being diagnosed with obesity by age of three years [48]. Numerous studies in South Africa have shown that early introduction of solids, even before two months, is a common practice [49,50,51].

Of some concern in the 1–<3-year-old group, particularly regarding overweight and obesity, is that more than 60% children had energy intakes greater than the EER. Even though this drops to only one in three in the older age groups, it remains a concern in terms of obesity risk. However, the EER results need to be interpreted conservatively as we used a physical activity level (PAL) for moderately active children, which may have under- or overestimated their energy needs. In all three age groups maize porridge was the main contributor to total energy intake (26%, 22% and 19%, respectively, from the youngest to oldest age group) and was also the most commonly consumed food item [52]. Maize porridge was also identified as being the most commonly consumed food item in the 1999 NFCS; as a result, it was selected as a food vehicle for fortification [53]. The high prevalence of maize intake in these studies (PDIS) and NFCS [54] may be linked to the high percentage of African black children included in the samples, reflecting the racial composition of the South African population. Maize porridge is a traditional staple food for black Africans in the country [54]. Further key contributors to total energy intake for all three groups were salty snacks (6–7%) and potato/sweet potato (5–6%). The prominence of potato/sweet potato as an energy source can possibly be explained by the fact that all potato/sweet potato containing items, irrespective of preparation, were included in this analysis.

Maize was also found to contribute the greatest amount to total carbohydrate intake (37%, 32% and 27%, respectively, from the youngest to the oldest age group), which was within or above the AMDRs for all children. Of concern is that the other key contributors to carbohydrate intake were mostly refined, i.e., granulated sugar in all three age groups, white bread in the two older age groups, white rice in the 3–<6-year-old group and sugar-sweetened cold drinks (carbonated and cordial/squash) in the 6–<10-year-olds. The remaining two of the four top carbohydrate sources in the 1–<3 age group were BMS and potato/sweet potato, the latter being a typical complementary food for infants [55]. It is evident from the results that the contribution of maize to total carbohydrate intake reduced somewhat with increasing age. Mean carbohydrate intake remained similar between 1999 [54] and 2018 in the different age groups, although it was somewhat higher in 2018. The quality of the main carbohydrate sources in the 1999 NFCS [54] was also largely poor, including granulated sugar, white bread, potatoes and white rice.

A serious concern is that the percentage contribution of free sugars to total energy was above the WHO cut-off of 10% for approximately half of the children in each of the three age groups (47%, 48% and 52%, respectively, from the youngest to the oldest age group). If the more conservative cut-off of %E greater than 5% of total energy recommended by the British Scientific Advisory Committee on Nutrition (SACN) [56] is considered, the situation is even more dire. Key contributors to free sugar intake in all three age groups include granulated sugar (40–42% of the children) that is typically added to tea, coffee and porridge [54], candies (19–20% of the children) and sugar-sweetened cold drinks (carbonated and cordial/squash) (8%, 11% and 18%, respectively, from the youngest to the oldest age group). Fruit does not feature as a source of free sugar or carbohydrate in general in any one of the age groups, despite it being amongst the 12 most frequently consumed food types (number 7 on the list).

Of note is that older children would be attending a preschool/crèche or primary school during weekdays, where they may have more ready access to sugary foods and drinks. A large majority of schools in South Africa sell unhealthy foods that include candies and cold drinks [16]. The main sources of added sugar in 1999 were granular sugar (mainly in tea/coffee), and cordial/squash [54]. There is a wealth of studies on a positive association between sugar sweetened beverages and obesity in children and adolescents [57,58,59], hence, it is disappointing to note that sugar intake has not decreased since 1999, despite the introduction of a sugar sweetened beverages levy [60], as well as numerous other efforts by the Department of Health to inform the public about the need to reduce sugar intake, including the food-based dietary guidelines [61] and other educational materials. The fact that most sugar is consumed as granular sugar means that further work needs to be done to address discretionary sugar consumption.

Bearing the above in mind, it comes as no surprise that the fibre intake of practically all the children was below the recommended intake level. The finding that maize was the main source of fibre in all three age groups (38%, 30% and 28%, respectively, from the youngest to oldest age group) is most probably linked to the fact that it was the main contributor to total energy and carbohydrate intake and provides between 1–3.5 g fibre per 100 g, depending on the consistency of the porridge [36]. Potato/sweet potato, which also featured as key contributor to total energy intake, was also one of the four top sources of fibre in all three age groups. Other sources of fibre were high fibre cereal (youngest two age groups), fresh fruit (1–<3-year-old group only), brown bread which contains 5.5 g fibre per 100 g (older two age groups) and white bread which contains 3.5 g fibre per 100 g [36] (oldest age group only). Even though vegetables were the third most commonly consumed foods in all three age groups, it was not sufficient enough to feature as a source of carbohydrate or fibre. Low intake of fruit and vegetables was reported in the 1999 NFCS [54], as well as the subsequent South African National Health and Nutrition Survey [62].

Protein intake was mostly in line with recommendations in all three age groups, with animal protein contributing more to total protein intake than plant protein, with the highest ratio of animal to plant protein in the youngest age group. Chicken, which is a more affordable animal protein than red meat, contributed most to total protein intake (g) in all three age groups (16–17% of the children) and was the 9th most commonly consumed food. Eggs, which are also a more affordable source of good quality protein, did not feature in the list of key contributors to protein or 12 most commonly consumed foods. Maize, a source of plant protein, was the second largest contributor to total protein intake in all three age groups (16%, 18% and 12%, respectively, from the youngest to oldest age group). A reduction in milk intake in the older children is evident: in the youngest age group’s whole milk intake was the third largest contributor to total protein intake, in the 3–<6-year-old group the fourth largest contributor, and not amongst the top four contributors in the oldest group. This profile is similar to the one recorded in 1999 in the NFCS [54]. While milk features in the list of commonly consumed items, the daily amounts consumed are very small, as frequently the milk is only added to tea or coffee [54].

A concern is the finding that more than half of 1–<3-year-old children had a total fat intake below the AMDR. This figure was 40% for the 3–< 6-year-old children and decreased to 5% for the 6–<10-year-old group. In fact, 26% of the oldest age group had an intake above the upper cut-off of the range, which could be considered a concern depending on the quality of fat consumed. The intake of adequate amounts of good quality fat was evident (mostly polyunsaturated and monounsaturated fats; less saturated and minimal trans fats are essential for growth and development, especially in younger children [52]. The quality of the fat consumed by the children seemed to be good, as PUFA intake was in or above the recommended range for more than 90% of children in the three age groups; saturated fat intake was <10% for 67%, 76% and 68% respectively from the youngest to the oldest group, while trans fats contributed 0.5–0.7% to total energy for the three age groups, which is in line with the recommendation of <1%. The mandatory maximum limits of trans fats in fats and oils intended for human consumption that were introduced in South Africa in 2011 [63] may have contributed to the low trans fat intake.

The food types that contributed most to total fat intake were salty snacks (10–11% of the children), chicken (8%, 9% and 8%, respectively, from the youngest to oldest age group), whole milk and BMS in the 1–<3-year-old group only (8% and 6% respectively), medium fat margarine in the two oldest groups (8–10%, respectively) and processed meats in these two groups (8–9% of the children). Sources of saturated fat across the three age groups included salty snacks, whole milk, processed meat, chicken and medium fat margarine. PUFA oil contributed 6%, 5% and 6%, respectively, from the youngest to the oldest age group to total fat intake of children. Fatty fish (e.g., pilchards) did not feature as a protein or fat source in any age group, despite being recommended as an affordable source of good quality protein and essential fatty acids [46].

Overall,, the mean total fat intakes as %E increased from 1999 (NFCS) to 2018 (PDIS) [15]. GTG and WC had a mean total fat intake of 27%E and 30%E, respectively, in 1999 [15]. In the PDIS, this increased from 30%E in the youngest group to 34%E in the oldest age group. Similar findings were noted for saturated fat intake. A recent review on the benefits of a reduced saturated fat intake indicated that there was a significant reduction in total cholesterol, LDL cholesterol and diastolic blood pressure [64]. These authors concluded that dietary guidelines should continue to recommend diets low in saturated fat for children.

A serious concern is the apparent high consumption of salty snacks, which include crisps and popcorn. These snacks are not only energy-dense and nutrient poor but have a high sodium content and may contain hydrogenated plant oils. It is known that a high sodium intake in children contributes to the development of hypertension later in life [65]. In South Africa in 2016, 46% women and 44% men were found to have hypertension [25]. Very likely sources of these snacks include tuck shops at schools [16] and informal food outlets in the community, such as street vendors or spaza (informal) shops [66]. Our results on the consumption of snack foods and sugar sweetened beverages are in line with findings by Pries et al., who conducted a systematic review on the contribution of snack food/sugar sweetened beverage consumption to total energy intake of children below 23 months of age in low-income countries [67]. The percent total energy from these items they reported ranged from 13% to 38%. However, they mention that at this stage associations with growth were found to be inconclusive and no studies assessed associations with nutrient intakes.

The most commonly consumed foods by children in the PDIS are reflective of a diet that places them at risk of NCD-related morbidity and mortality according to the Global Burden of Disease Study Collaborators [68]. They found that key dietary risk factors for these diseases are diets low in whole grains, fruit, vegetables, nuts, seeds, omega-3 fatty acids and high in sodium. Items such as granulated sugar, salty snacks, cold drinks and candies should not form the basis of the diets of children and nor should the main forms of carbohydrates be refined. Of further concern is that key changes between the commonly eaten foods in 1999 and 2018 are that salty snacks and candies now form part of the top 12 items, intake of sugar and sweetened cold drinks remain high, and fruit and vegetables were consumed by less than 50% of children.

Predictors of low energy, low protein, low and high fat intake and high intake of free sugars were similar for most variables. Important significant predictors were frequently dependent on who the household head was, the province lived in, and food security status, while a significant protective factor was the mother having completed high school. The latter emphasizes the importance of girls completing high school.

One of the limitations of the study is that the PDIS was conducted in only two of the nine provinces, and these were both urbanized; no significant urban rural differences were found within the provinces. The information cannot therefore be extrapolated to other provinces that are less urbanized, nor to the deep rural areas. The accuracy of the data presented is not only reliant on the method used to assess dietary intake, but also the availability of food composition data that reflect the true nutrient composition of the foods consumed. As with many other countries, the local food composition tables rely on food composition from other countries, where local data are not available, and these tables are updated per food group periodically and in this particular study, do not reflect the changes that have been brought about by a number of regulations aimed at reducing nutrients in foods (such as trans fats, sugar). These factors have been accounted for as far as possible, yet gaps remain. Furthermore, despite trying to be as accurate as possible when doing dietary recalls, it should be kept in mind that the primary caregivers may not always have been with the children during the previous 24 h. Another difficulty was the fact that the RDAs were used in 1999, since the DRIs were not yet available, while the DRIs have been used in the PDIS, making some comparisons difficult.

## 5. Conclusions

The children in the two provinces studied showed evidence of an advanced nutrition transition that places them at risk of morbidity and mortality of numerous NCDs. This includes having a diet high in salty foods, free sugars, saturated fat and low in dietary fibre. The diet contains many refined and processed foods and shows little dietary diversity, including a low intake of fruit and vegetables and nuts and seeds. The finding that mean intakes were similar between urban and rural areas further supports the finding that a nutrition transition has taken place in these two provinces.

## Figures and Tables

**Table 1:** Recommended cut-off values for dietary variables.

Variables	Recommended Values	Reference
Energy intake kJ	Estimated energy requirement (EER) for healthy moderately active children: (kJ/day) M (1–2 yrs): 4393 kJ; F (1–2 yrs): 4166 kJ; M (3 yrs): 6213 kJ; F (3 yrs): 5837 kJ; M (4 yrs): 6552 kJ; F (4 yrs): 6171 kJ; M (5 yrs): 6937 kJ; F (5 yrs): 6514 kJ; M (6 yrs): 7289 kJ; F (6 yrs): 6870 kJ; M (7 yrs): 7699 kJ; F (7 yrs): 7192 kJ; M (8 yrs): 8079 kJ; F (8 yrs): 7573 kJ; M (9 yrs): 8548 kJ; F (9 yrs): 7908 kJ	[37]
Carbohydrate	Acceptable macronutrient distribution range (AMDR) 45–65%	[37]
Total fat	AMDR: %E from fat in 1–3-year-olds 30–40%, 4–9 -year-olds 25–35%	[38]
Saturated fat	<10% of energy	[38]
Free sugars	<10% of energy	[39]
Fibre	Adequate intake (AI) AI: 1–3-year-olds −13 g, 4–8-year-olds 25 g, boys 9 years 31 g, girls 9 years 29 g	[37]

**Table 2:** Sociodemographic and other characteristics of the 1–<10-year-old children in the two provinces studied.

	Gauteng N = 733 % (95% CI)	Western Cape N = 593 % (95% CI)	Rao–Scott Chi Square *p* Values	All N = 1326 % (95% CI)
Primary caregiver				
Mother	70.1 (65.6–74.6)	71.0 (64.7–77.2)	0.0448 *	70.4 (66.8–74.0)
Father	6.6 (3.4–9.7)	1.8 (0.2–3.3)		5.0 (2.8–7.1)
Grandparent	16.7 (12.9–20.4)	21.0 (15.5–26.4)		18.1 (15.0–21.2)
Other (e.g., sibling, aunt)	6.7 (4.0–9.5)	6.3 (2.1–10.4)		6.6 (4.3–8.8)
**Age in Years**				
1–<3-years	26.3 (22.1–30.6)	25.3 (19.4–31.2)	0.9234	26.0 (22.6–29.4)
3–<6-years	35.4 (31.0–39.8)	35.1 (30.7–39.5)		35.3 (32.1–38.5)
6–<10-years	38.3 (34.1–42.4)	39.6 (33.1–46.1)		38.7 (35.2–42.2)
Gender				
Male	50.2 (45.5–54.9)	47.5 (43.1–51.9)	0.3907	49.3 (45.9–52.7)
Female	49.8 (45.1–54.5)	52.5 (48.1–56.9)		50.7 (47.3–54.1)
**Head of Household**				
Father	40.2 (33.8–46.6)	38.8 (34.6–43.0)	0.1320	39.7 (35.3–44.1)
Mother	16.8 (13.8–19.9)	10.8 (7.0–14.5)		14.8 (12.5–17.2)
Grandmother	21.9 (15.5–28.3)	28.3 (21.8–34.9)		24.0 (19.3–28.8)
Grandfather	11.7 (8.3–15.1)	14.0 (10.0–18.0)		12.5 (9.9–15.0)
Other (e.g., aunt, uncle)	9.4 (5.7–13.1)	8.1 (4.9–11.4)		9.0 (6.3–11.7)
**Marital Status of Mother**				
Unmarried	41.1 (34.9–47.2)	34.8 (28.4–41.1)	0.0002 **	39.0 (34.4–43.5)
Married	24.9 (20.5–29.4)	41.3 (33.3–49.2)		30.4 (26.4–34.3)
Divorced/widowed	4.8 (2.5–7.0)	2.4 (0.7–4.2)		4.0 (2.4–5.6)
Living together	27.8 (22.0–33.6)	20.8 (15.9–25.7)		25.5 (21.4–29.6)
Other	1.4 (0.2–2.6)	0.8 (0.0–1.8)		1.2 (0.3–2.1)
**Mother’s Highest Education**				
Not completing Gr. 12	51.2 (44.9–57.4)	57.7 (47.1–68.3)	0.1833	53.3 (47.9–58.7)
Completion of Gr. 12	33.9 (28.4–39.4)	24.7 (17.6–31.8)		30.8 (26.5–35.2)
Qualification after Gr.12	12.2 (8.7–15.7)	15.6 (7.6–23.6)		13.3 (9.9–16.8)
Do not know	2.8 (1.4–4.1)	2.0 (0.5–3.5)		2.5 (1.5–3.5)
**Father’s Highest Education**				
Not completing Gr. 12	26.9 (22.0–31.7)	33.8 (29.0–38.5)	0.3232	29.1 (25.6–32.7)
Completion of Gr. 12	32.6 (26.9–38.3)	30.4 (25.2–35.6)		31.9 (27.8–36.0)
Qualification after Gr.12	13.1 (9.4–16.9)	10.7 (5.7–15.7)		12.3 (9.4–15.3)
Do not know	27.4 (22.4–32.4)	25.2 (19.7–30.6)		26.7 (22.9–30.4)
**Mother’s Employment Status**				
Yes	22.4 (17.8–26.9)	38.4 (31.0–45.9)	<0.0001 ***	27.7 (23.9–31.5)
No	74.6 (69.6–79.6)	60.2 (53.0–67.5)		69.8 (65.8–73.9)
Do not know/not applicable	3.0 (1.3–4.7)	1.3 (0.3–2.4)		2.5 (1.3–3.6)
**Father’s Employment Status (%)**				
Yes	64.8 (60.6–69.1)	65.3 (59.7–70.9)	0.9532	65.0 (61.6–68.4)
No	21.4 (17.5–25.3)	20.5 (15.1–25.9)		21.1 (18.0–24.2)
Do not know/not applicable	13.8 (11.1–16.4)	14.1 (10.2–18.1)		13.9 (11.7–16.1)
**Wealth Index Quintiles**				
One	21.1 (14.6–27.6)	17.7 (10.7–24.7)	0.2633	20.0 (15.1–24.8)
Two	17.8 (12.0–23.6)	24.3 (20.0–28.6)		20.0 (15.9–24.0)
Three	21.3 (17.0–25.7)	17.0 (12.6–21.4)		19.9 (16.7–23.1)
Four	21.5 (16.7–26.3)	17.5 (12.4–22.6)		20.2 (16.6–23.7)
Five	18.3 (11.6–25.0)	23.5 (14.5–32.5)		20.0 (14.7–25.3)
**Ethnicity**				
Black African	97.8 (96.0–99.6)	27.6 (12.9–42.3)	<0.0001	74.5 (69.5–79.4)
Mixed ancestry	2.2 (0.3–4.0)	68.0 (53.7–82.4)		24.1 (19.2–28.9)
Other	0.0 (0.0–0.1)	4.4 (0.6–8.2)		1.5 (0.3–2.7)
**Type of Residence**				
Rural	2.4 (0.7–4.1)	6.6 (1.6–11.5)	0.1938	3.8 (1.9–5.7)
Urban formal	88.9 (82.3–95.4)	86.8 (79.1–94.5)		88.2 (83.2–93.2)
Urban informal	8.7 (2.7–14.7)	6.6 (1.7–11.5)		8.0 (3.7–12.3)
**Mother’s BMI [39]**				
Underweight/normal BMI ≤ 18.5 & 18.5–24.9 kgm^2^	33.3 (28.0–38.5)	29.1 (23.6–34.5)	0.0023 **	32.0 (28.0–35.9)
Overweight BMI = 25–29.9 kgm^2^	27.7 (23.6–31.8)	20.4 (16.5–24.3)		25.4 (22.4–28.5)
Obese BMI ≥ 30 kgm^2^	39.1 (35.8–42.3)	50.6 (43.0–58.1)		42.6 (39.4–45.8)
**Hunger Scale [25]**				
Total score = 0: No risk	57.9 (49.5–66.3)	48.8 (38.9–58.7)	0.1483	54.9 (48.5–61.3)
1–4: At risk of hunger	22.1 (17.2–27.0)	28.9 (23.0–34.9)		24.4 (20.6–28.2)
5–8: Food shortage in house	20.0 (14.8–25.1)	22.3 (16.5–28.0)		20.7 (16.8–24.6)

Gr.: grade, * *p* < 0.05; ** *p* < 0.01; *** *p* < 0.0001.

**Table 3:** The mean energy (95%CI), carbohydrate, protein, and fibre intake of children aged 1–<10-years by age and province (N = 1326).

Age (years)		1–<3			3–<6			6–<10	
Province	GTG	WC	All	GTG	WC	All	GTG	WC	All
Sample size	185	148	333	282	232	514	266	213	479
Total energy (kJ)	4813 (4366–5260)	5220 (4105–6335)	4944 (4465–5424)	5389 * (4847–5931)	6105 (5860–6350)	5626 (5264–5987)	6537 (6340–6735)	6515 (6150–6880)	6530 (6329–6730)
Carbohydrate (g)	165.1 (156.2–173.9)	160.7 (114.9–206.5)	163.7 (148.7–178.6)	182.0 * (172.5–191.6)	197.9 (192.6–203.2)	187.3 (181.5–193.1)	214.0 ** (208.6–219.4)	199.3 (195.0–203.6)	209.0 (205.0–213.0)
%E from carbohydrate	60.5 * (58.8–62.2)	54.3 (49.6–59.0)	58.5 (55.9–61.0)	61.2 * (59.0–63.3)	57.9 (56.5–59.3)	60.1 (58.6–61.5)	58.5 * (57.5–59.5)	54.5 (52.0–57.0	57.1 (56.0–58.3)
Total protein(g)	32.0 * (27.5–36.5)	40.2 (35.7–44.7)	34.6 (30.9–38.4)	37.5 ** (32.9–42.1)	46.1 (45.6–46.7)	40.3 (37.4–43.2)	44.9 (41.6–48.2)	48.7 (43.0–54.4)	46.2 (43.0–49.4)
%E from protein	11.2 * (10.5–11.9)	13.1 (11.6–14.5)	11.8 (10.9–12.7)	11.8 * (11.6–12.1)	12.9 (12.2–13.6)	12.2 (11.8–12.5)	11.7 * (11.5–12.0)	12.8 (11.9–13.7)	12.1 (11.7–12.4)
Animal protein(g)	15.3 ** (13.4–17.2)	23.2 (20.4–26.0)	17.8 (15.4–20.3)	18.7 ** (15.8–21.6)	25.9 (24.8–27.0)	21.0 (19.5–22.6)	22.1 * (19.8–24.4)	28.1 (23.7–32.4)	24.1 (21.8–26.5)
%E from animal protein	5.6 (4.9–6.4)	7.8 (5.2–10.5)	6.3 (4.9–7.7)	5.9 ** (5.7–6.2)	7.3 (6.5–8.1)	6.4 (6.0–6.7)	5.9 ** (5.5–6.2)	7.5 (6.7–8.3)	6.4 (6.0–6.8)
Plant protein (g)	13.2 ** (12.8–13.5)	10.4 (9.2–11.5)	12.3 (11.6–12.9)	17.8 ** (16.5–19.1)	15.0 (14.7–15.4)	16.9 (15.9–17.9)	21.9 *** (21.6–22.2)	18.7 (18.0–19.3)	20.8 (20.5–21.2)
%E from plant protein	4.6 ** (4.2–5.0)	3.3 (2.9–3.8)	4.2 (3.9–4.6)	5.6 *** (5.4–5.8)	4.2 (3.9–4.4)	5.1 (4.9–5.3)	5.6 ** (5.3–5.8)	4.7 (4.6–4.8)	5.3 (5.2–5.4)
Fibre (g)	10.8 * (10.5–11.1)	8.7 (7.0–10.5)	10.1 (9.4–10.9)	13.0 (11.9–14.1)	12.2 (12.0–12.3)	12.7 (12.0–13.4)	14.7 ** (14.2–15.3)	12.8 (12.3–13.3)	14.1 (13.6–14.5)

* *p* < 0.05; ** *p* < 0.01; *** *p* < 0.0001 Independent t-test for differences between provincial means; %E: Percent of energy intake; EER = Estimated energy requirement [37]: M (1–2 yrs): 4393 kJ; F (1–2 yrs): 4166 kJ; M (3 yrs): 6213 kJ; F (3 yrs): 5837 kJ; M (4 yrs): 6552 kJ; F (4 yrs): 6171 kJ; M (5 yrs): 6937 kJ; F (5 yrs): 6514 kJ; M (6 yrs): 7289 kJ; F (6 yrs): 6870 kJ; M (7 yrs): 7699 kJ; F (7 yrs): 7192 kJ; M (8 yrs): 8079 kJ; F (8 yrs): 7573 kJ; M (9 yrs): 8548 kJ; F (9 yrs): 7908 kJ; Acceptable macronutrient distribution range (AMDR): Carbohydrate %E: 45–65%, [37] %E from protein in 1-3-year-olds: 5–20%, 4–9-year-olds 10–30% [37]. Protein RDA 1–3-year-olds 13 g, 4–8-year-olds 19 g, 9-year-olds 34 g [37]. Total fibre adequate intake (AI) [37] 1–3-year-olds −13 g, 4–9-year-olds 25 g, and free sugars intake <10%E [39].

**Table 4:** The mean (95% CI) fat and free sugars intake of children aged 1–<10-years by province and age group.

Age (years)		1–<3			3–<6			6–<10	
Province	GTG	WC	All	GTG	WC	All	GTG	WC	All
Sample size	185	148	333	282	232	514	266	213	479
Total fat	36.7 * (31.5–41.9)	45.1 (41.3–48.8)	39.4 (34.7–44.0)	41.1 (33.9–48.4)	49.7 (45.0–54.4)	44.0 (38.8–49.1)	54.9 (52.0–57.9)	59.0 (52.9–65.0)	56.3 (53.2–59.4)
%E fat	29.0 (28.2–29.7)	33.2 (28.4–38.1)	30.3 (28.7–32.0)	28.1 (26.5–29.7)	30.0 (27.9–32.0)	28.7 (27.6–29.9)	31.4 * (30.6–32.2)	33.8 (31.9–35.8)	32.2 (31.3–33.2)
Total SF (g)	10.5 ** (8.4–12.7)	14.9 (14.4–15.5)	12.0 (10.0–13.9)	11.9 * (8.8–15.0)	15.6 (13.6–17.7)	13.1 (10.8–15.5)	14.8 ** (13.8–15.9)	17.9 (16.6–19.2)	15.9 (15.0–16.7)
%E from SF	8.1 (7.2–9.0)	10.6 (8.1–13.2)	8.9 (7.5–10.3)	8.1 * (7.2–9.0)	9.4 (8.4–10.4)	8.5 (7.9–9.1)	8.5 ** (8.2–8.9)	10.3 (10.1–10.6)	9.1 (8.9–9.4)
Cholesterol (mg)	83.9 ** (60.6–107.2)	154.8 (118.0–191.5)	106.8 (82.5–131.2)	115.4 (86.2–144.6)	146.9 (130.8–163.0)	125.8 (105.7–146.0)	139.7 ** (126.5–152.9)	178.7 (164.8–192.7)	153.0 (145.5–160.4)
MUFA (g)	10.6 * (9.3–11.9)	13.1 (11.2–15.0)	11.4 (10.0–12.8)	12.9 (10.2–15.6)	15.3 (14.0–16.7)	13.7 (11.9–15.5)	16.9 ** (16.3–17.4)	19.5 (18.1–20.8)	17.7 (17.1–18.4)
%E MUFA	8.6 (8.2–9.0)	9.8 (6.3–13.4)	9.0 (7.7–10.3)	8.8 (8.1–9.5)	9.2 (8.7–9.8)	9.0 (8.6–9.3)	9.7 ** (9.4–10.0)	11.2 (10.9–11.6)	10.2 (9.9–10.5)
PUFA	9.6 (8.7–10.6)	9.4 (8.9–9.80	9.5 (8.8–10.3)	12.3 (11.7–12.9)	12.0 (11.5–12.6)	12.2 (11.9–12.5)	17.7 (16.3–19.1)	16.2 (13.5–18.8)	17.2 (15.9–18.5)
%E PUFA	8.0 (6.6–9.5)	7.2 (5.6–8.7)	7.8 (6.7–8.8)	8.4 ** (7.9–9.0)	7.3 (7.1–7.4)	8.1 (7.7–8.5)	9.9 (9.4–10.5)	9.0 (7.7–10.3)	9.6 (9.0–10.2)
Trans fats (g)	0.7 (0.4–1.0)	1.0 (0.7–1.3)	0.8 (0.5–1.1)	0.8 ** (0.7–1.0)	1.2 (1.1–1.2)	0.9 (0.9–1.0)	1.0 * (1.0–1.1)	1.4 (1.1–1.7)	1.2 (1.0–1.3)
%E Trans fats	0.5 (0.3–0.7)	0.7 (0.3–1.0)	0.6 (0.3–0.8)	0.6 ** (0.5–0.7)	0.7 (0.7–0.8)	0.6 (0.6–0.7)	0.6 *(0.5–0.6)	0.8 (0.6–0.9)	0.7 (0.6–0.7)
Free sugars (g)	29.6 (25.9–33.2)	33.8 (28.9–38.7)	30.9 (27.5–34.4)	32.4 (26.2–38.7)	36.9 (35.9–37.9)	33.9 (30.0–37.9)	39.0 (34.9–43.1)	42.6 (36.9–48.3)	40.2 (37.0–43.5)
%E Free sugars	9.9 (8.5–11.3)	10.3 (7.8–12.9)	10.0 (8.6–11.4)	10.1 (9.6–10.7)	10.2 (9.0–11.4)	10.2 (9.5–10.8)	10.0 (8.7–11.3)	11.0 (8.4–13.5)	10.3 (8.9–11.8)

SF: Saturated fat; MUFA: Monounsaturated fatty acids, PUFA: Polyunsaturated fatty acids, %E: Percent of energy intake; * *p* < 0.05; ** *p* < 0.01; *** *p* < 0.0001 Independent *t*-test for differences between provincial means; %E from fat in 1–3-year-olds 30–40%, 4–9-year-olds 25–35% [38], saturated fat is <10%E [38]; free sugars <10%E [39].

**Table 5:** The four main foods contributing to total energy and macronutrient intake of 1–<10-year-olds in both provinces by age group.

1–<3 -Years- Old (N = 333)			3–<6 -Years-Old (N = 514)			6–<10-Years-Old (N = 479)		
Food Contributing to kJ	Contribution to KJ Per Capita	% of total kJ	Food Contributing to KJ	Contribution to KJ Per Capita g	% of Total KJ	Food Contributing to KJ	Contribution to KJ Per Capita g	% of Total kJ
Maize porridge	1265.3	25.6	Maize porridge	1248.6	22.3	Maize porridge	1206.5	18.5
Salty snacks	275.3	5.6	Potato/sw. potato	324.2	5.8	White bread	466.7	7.2
Potato/sw. potato	238.4	4.8	Salty snacks	323.2	5.8	Salty snacks	423.1	6.5
Whole milk	236.6	4.8	Chicken	293.9	5.2	Potato/sw. potato	370.9	5.7
**Food Contributing to CHO**	**Contribution to CHO Per Capita g**	**% of Total CHO**	**Food Contributing to CHO**	**Contribution to CHO Per Capita g**	**% of Total CHO**	**Food Contributing to CHO**	**Contribution to CHO Per Capita g**	**% of Total CHO**
Maize porridge	60.18	36.8	Maize porridge	59.4	32.0	Maize porridge	56.9	27.0
Granulated sugar	10.13	6.2	Granulated sugar	12.9	7.0	White bread	20.5	9.7
Potato/sw. potato	7.35	4.5	White bread	10.8	5.8	Granulated sugar	14.5	6.9
BMS	6.53	4.0	White rice	10.4	5.6	Cold drink	11.8	5.6
**Food Contributing to Protein**	**Contribution to Protein (Per Capita g)**	**% of Total Protein**	**Food Contributing to Protein**	**Contribution to Protein (Per Capita g)**	**% of Total Protein**	**Food Contributing to Protein**	**Contribution to Protein (Per Capita g)**	**% of Total Protein**
Chicken	5.9	16.6	Chicken	7.8	16.6	Chicken	7.6	16.1
Maize porridge	5.6	15.7	Maize porridge	5.5	17.7	Maize porridge	5.5	11.6
Whole milk	2.9	8.2	Beef	2.5	8.2	White bread	4.0	8.4
Beef	1.8	5.2	Whole milk	2.3	5.2	Beef	3.4	7.1
**Food Contributing to Fat**	**Contribution to Fat (Per Capita g)**	**% Total Fat**	**Food Contributing to Fat**	**Contribution to Fat (Per Capita g)**	**% Total Fat**	**Food Contributing to Fat**	**Contribution to Fat (Per Capita g)**	**% Total Fat**
Salty snacks	4.1	10.4	Salty snacks	4.8	10.8	Salty snacks	6.2	11.4
Whole milk	3.1	7.9	Chicken	4.0	9.0	Med fat margarine	5.7	10.4
Chicken	3.1	7.8	Med fat margarine	3.7	8.3	Processed meat	4.8	8.7
BMS	2.4	6.3	Processed meat	3.5	8.0	Chicken	4.1	7.5
**Foods Contributing to Saturated Fat**	**Contribution to SF (Per Capita g)**	**% of total SF**	**Foods Contributing to Saturated Fat**	**Contribution to SF (Per Capita g)**	**% of SF**	**Foods Contributing to Saturated Fat**	**Contribution to SF (Per Capita g)**	**% of SF**
Whole milk	1.8	14.5	Salty snacks	1.7	12.7	Salty snacks	2.2	13.9
Salty snacks	1.4	11.9	Whole milk	1.4	10.6	Processed meat	1.7	11.2
Processed meat	0.8	6.9	Processed meat	1.3	9.8	Whole milk	1.3	8.3
Chicken	0.8	6.5	Chicken	1.0	7.9	Med fat margarine	1.2	7.4
**Foods Contributing to Added Sugar**	**Contribution to AS (Per Capita g)**	**% of Total AS**	**Foods Contributing to Added Sugar**	**Contribution to AS (Per Capita g)**	**% of AS**	**Foods Contributing to AS**	**Contribution to AS (Per Capita g)**	**% of AS**
Granulated sugar	10.1	39.6	Granulated sugar	12.9	42.0.4	Granulated sugar	14.5	39.7
Candy	4.7	18.5	Candy	6.1	19.9	Candy	6.8	18.6
Cold drinks	2.1	8.3	Cold drinks	3.3	10.7	Cold drinks	6.5	17.7
Yoghurt	1.9	7.3	Cookies	1.7	5.4	Cookies	2.2	6.0
**Foods Contributing to fibre**	**Contribution to Fibre (Per Capita g)**	**% of Total Fibre**	**Foods Contributing to Fibre**	**Contribution to Fibre (Per Capita g)**	**% of Total Fibre**	**Foods Contributing to Fibre**	**Contribution to Fibre (Per Capita g)**	**% of Total Fibre**
Maize porridge	3.8	37.9	Maize porridge	3.7	30.2	Maize porridge	3.8	27.5
High fibre cereal	1.1	10.5	High fibre cereal	1.3	10.3	White bread	1.4	10.2
Potato/sw. potato	0.7	7.1	Brown bread	1.0	7.9	Brown bread	1.4	9.7
Fresh fruit	0.5	5.3	Potato/sw. potato	0.9	7.4	Potato/sw. potato	1.1	7.7

PUFA oil contributed 6%, 5% and 6% respectively, from the youngest to the oldest age group to total fat intake. CHO: Carbohydrate; SF: Saturated fat; Salty snacks: crisps and popcorn; Chicken, beef, potatoes: only the item was indicated, not the cut or method of cooking; Cold drinks: sugar-sweetened cold drinks and cordial/squash; BMS: breast milk substitute/formula; sw: sweet; Maas: a sour drink made from maize; Fresh fruit: not vitamin A or vitamin C rich; Med: medium.

**Table 6:** Children still receiving breast milk by age group and province.

	Gauteng		Western Cape		All	
	1–< 3- Years	3–<6-Years	1–<3- Years	3–< 6- Years	1–<3- Years	3–<6- Years
Number of children sampled	185	282	148	232	333	514
Number breastfeeding	17	1	22	4	39	5
% Infants breastfed	9.2	0.4	14.9	1.7	11.7	1.0
Mean (SD) intake of breast milk per day(g) (only consumers)	335.3 (177)	200.0 (-)	272.7 (172)	175.0 (96)	300.0 (175)	180.0 (84)
Mean (SD) frequency of feeds	3.4 (1.8)	2.0 (-)	2.7 (1.7)	1.8 (1.0)	3.0 (1.7)	1.8 (0.8)
% Contribution to energy intake (consumers of breast milk only)	21.4	8.8	16.7	7.8	18.7	8.0
Mean (SD) kJ portion p/day	995.8 (524)	594.0 (-)	810.0 (512)	519.8 (284)	891.0 (519)	534.6 (249)

**Table 7:** Bivariate and multivariate logistic regression of sociodemographic factors on having an energy intake below the estimated energy intake (EER) by age group.

	Bivariate Logistic Regression			Multivariate Logistic Regression (Adjusted for Gender and Ethnicity)		
	Children 1–<3-Yrs Energy < EER N = 333 (n = 145) OR (95% CI)	Children 3–<6-Yrs Energy < EER N = 514 (n = 349) OR (95% CI)	Children 6–<10-Yrs Energy < EER N = 479 (n = 351) OR (95% CI)	Children 1–<3-Yrs Energy < EER N = 333 (n = 145) OR (95% CI)^b^	Children 3–<6-Yrs Energy < EER N = 514 (n = 349) OR (95% CI)^c^	Children 6–<10-Yrs Energy < EER N = 479 (n = 351) OR (95% CI) ^d^
Primary caregiver ^a^						
Mother	Ref	Ref	Ref			
Grandparent	1.50 (0.59–3.84)	1.50 (0.76–2.96)	1.72 (0.82–3.62)			
Other (e.g., father, aunt)	1.40 (0.46–4.20)	2.13 (0.89–5.08)	1.37 (0.67–2.79)			
Gender						
Male	Ref	Ref	Ref	Ref	Ref	Ref
Female	0.75 (0.45–1.25)	0.77 (0.49–1.23)	0.69 (0.41–1.18)	0.64 (0.37–1.11)	0.74 (0.47–1.17)	0.66 (0.38–1.16)
Head of household						
Father	Ref	Ref	Ref	Ref		
Mother	1.11 (0.34–3.60)	0.65 (0.32–1.33)	0.90 (0.42–1.95)	0.90 (0.27–2.96)		
Grandparent	1.93 (1.05–3.56) *	0.95 (0.57–1.58)	0.69 (0.40–1.21)	1.89 (0.94–3.82)		
Other (e.g., aunt, uncle)	3.82 (1.19–12.25) *	0.82 (0.36–1.85)	0.75 (0.24–2.36)	4.77 (1.63–13.91) **		
Marital status of mother						
Married	Ref	Ref	Ref			
Other (e.g., unmarried, divorced)	1.67 (0.77–3.64)	1.11 (0.63–1.97)	1.06 (0.56–1.98)			
Mother’s highest education						
Did not complete grade 12	Ref	Ref	Ref	Ref		Ref
Completed grade 12	0.33 (0.17–0.62) **	1.33 (0.79–2.26)	0.44 (0.25–0.75) **	0.27 (0.14–0.51) **		0.52 (0.29–0.92) *
Qualification after grade 12	0.27 (0.09–0.81) *	1.05 (0.58–1.91)	0.79 (0.38–1.68)	0.26 (0.08–0.91) *		0.84 (0.34–2.03)
Father’s highest education						
Did not complete grade 12	Ref	Ref	Ref			
Completed grade 12	0.62 (0.31–1.24)	0.87 (0.49–1.54)	0.76 (0.36–1.59)			
Qualification after grade 12	0.39 (0.13–1.24)	0.66 (0.31–1.41)	0.50 (0.23–1.11)			
Mother’s employment status						
Not employed/do not know	Ref	Ref	Ref			
Employed	0.47 (0.22–1.01)	1.06 (0.65–1.73)	1.44 (0.80–2.61)			
Father’s employment status						
Not employed/do not know	Ref	Ref	Ref			
Employed	0.73 (0.37–1.42)	1.06 (0.59–1.89)	0.78 (0.42–1.44)			
Wealth index quintiles						
One/two/three	Ref	Ref	Ref			Ref
Four/five	0.74 (0.39–1.41)	0.67 (0.38–1.18)	0.56 (0.35–0.89) *			0.73 (0.41–1.28)
Ethnicity						
Black African	Ref	Ref	Ref	Ref	Ref	Ref
Mixed Ancestry	1.06 (0.52–2.16)	0.61 (0.34–1.11)	1.40 (0.66–2.98)	1.21 (0.59–2.48)	0.63 (0.33–1.20)	1.35 (0.62–2.93)
Province						
Western Cape	Ref	Ref	Ref			
Gauteng	1.77 (0.91–3.47)	1.63 (0.92–2.90)	0.75 (0.44–1.27)			
Type of residence						
Urban formal	Ref	Ref	Ref			
Urban informal	1.32 (0.65–2.69)	1.45 (0.77–2.73)	0.61 (0.34–1.08)			
Rural	1.18 (0.62–2.25)	1.00 (0.57–1.76)	0.91 (0.55–1.49)			
Mother’s BMI						
Underweight/normal weight	Ref	Ref	Ref			Ref
Overweight	0.78 (0.34–1.79)	0.99 (0.58–1.68)	1.04 (0.45–2.39)			1.28 (0.53–3.09)
Obese	0.53 (0.25–1.15)	0.99 (0.56–1.76)	0.45 (0.22–0.90) *			0.55 (0.26–1.18)
Hunger scale						
Total score = 0: No risk	Ref	Ref	Ref		Ref	
1–4: At risk of hunger	0.73 (0.37–1.45)	0.54 (0.34–0.87) *	1.14 (0.55–2.38)		0.54 (0.33–0.86) *	
5–8: Food shortage in house	1.61 (0.63–4.12)	0.56 (0.33–0.93) *	1.05 (0.50–2.20)		0.53 (0.32–0.88) *	

^a^ Primary caregiver: person who looks after child most of the time; OR: odds ratio; * Odds ratio significant, *p* < 0.05; ** Odds ratio significant, *p* < 0.01; *** Odds ratio significant, *p* < 0.0001; N-values reflect the total number of children, n represents the number of children in the risk group.^b^ Children 1–<3-years: Estimate the risk of head of household and mother’s highest level of education for energy < EER, adjusting for ethnicity and gender. ^c^ Children 3–<6years: Estimate the risk using the hunger scale for energy < EER, adjusting for ethnicity and gender. ^d^ Children 6–<10-years: Estimate the risk of the mother’s highest level of education, the risk according to the wealth index quintiles and mother’s BMI for energy < EER, adjusting for ethnicity and gender.

**Table 8:** Bivariate and multivariate logistic regression of sociodemographic factors on having a protein intake below the acceptable macronutrient distribution range (AMDR) by age group.

	Bivariate Logistic Regression			Multivariate Logistic Regression (Adjusted for Gender and Ethnicity)		
	Children 1–<3-Yrs %E from Protein < AMDR N = 333 (n = 5) OR (95% CI) Nothing Significant	Children 3–<6-Yrs %E from Protein < AMDR N = 514 (n = 89) OR (95% CI)	Children 6–<10-Yrs %E from Protein < AMDR N = 479 (n = 131) OR (95% CI)	Children 1–<3-Yrs %E from Protein< AMDR N = 333 (n = 5) OR (95% CI) Nothing Significant	Children 3–<6-Yrs %E from Protein< AMDR N = 514 (n = 89) OR (95% CI) ^b^	Children 6–<10-Yrs %E from Protein< AMDR N = 479 (n = 131) OR (95% CI) ^c^
**Primary Caregiver ^a^**						
Mother		Ref	Ref			
Grandparent		0.70 (0.33–1.47)	0.84 (0.43–1.66)			
Other (father, sibling, aunt)		0.60 (0.21–1.72)	1.31 (0.71–2.42)			
Age in years						
Gender						
Male		Ref	Ref		Ref	Ref
Female		0.98 (0.60–1.60)	0.86 (0.49–1.49)		1.06 (0.63–1.77)	0.74 (0.42–1.33)
Head of household						
Father		Ref	Ref		Ref	
Mother		2.50 (1.15–5.44) *	2.03 (0.97–4.25)		2.38 (1.10–5.17) *	
Grandparent		1.02 (0.46–2.25)	1.10 (0.62–1.95)		1.05 (0.46–2.42)	
Other (e.g., aunt, uncle, friend)		1.04 (0.37–2.91)	2.12 (0.95–4.73)		1.02 (0.36–2.90)	
Marital status of mother						
Married		Ref	Ref			
Other e.g., unmarried, divorced		1.21 (0.73–2.02)	1.32 (0.71–2.48)			
Mother’s highest education						
Did not complete grade 12		Ref	Ref		Ref	
Completed grade 12		0.58 (0.34–1.00) *	0.96 (0.56–1.64)		0.54 (0.30–0.96) *	
Qualification after grade 12		0.80 (0.36–1.78)	0.75 (0.35–1.62)		0.91 (0.41–2.04)	
Father’s highest education						
Did not complete grade 12		Ref	Ref			
Completed grade 12		1.08 (0.62–1.89)	0.97 (0.61–1.52)			
Qualification after grade 12		0.94 (0.40–2.19)	0.82 (0.34–1.98)			
Mother’s employment status						
Not employed/do not know		Ref	Ref			
Employed		0.88 (0.46–1.69)	0.70 (0.38–1.31)			
Father’s employment status						
Not employed/do not know		Ref	Ref			
Employed		0.85 (0.48–1.51)	1.08 (0.67–1.75)			
Wealth index quintiles						
One/two/three		Ref	Ref			
Four/five		1.46 (0.81–2.64)	0.80 (0.49–1.30)			
Ethnicity						
Black African		Ref	Ref		Co-linear with province	Co-linear with province
Mixed ancestry		0.64 (0.30–1.34)	0.55 (0.31–0.98) *			
Province						
Western Cape		Ref	Ref		Ref	Ref
Gauteng		2.03 (1.10–3.77) *	2.25 (1.30–3.90) **		2.07 (1.08–4.00) *	2.23 (1.33–3.72) **
Type of residence						
Urban formal		Ref	Ref			
Urban informal		1.44 (0.86–2.43)	1.19 (0.73–1.94)			
Rural		0.60 (0.28–1.26)	0.47 (0.24–0.92)			
Mother’s BMI						
Underweight/normal weight		Ref	Ref			Ref
Overweight		0.61 (0.27–1.40)	2.53 (1.15–5.54) *			2.30 (1.06–5.01) *
Obese		0.96 (0.48–1.90)	2.15 (1.13–4.07) *			2.23 (1.18–4.24) *
Hunger scale						
Total score = 0: No risk		Ref	Ref			
1–4: At risk of hunger		1.53 (0.77–3.03)	1.09 (0.54–2.22)			
5–8: Food shortage in house		1.52 (0.83–2.78)	1.86 (0.89–3.89)			

^a^ Primary caregiver: person who looks after child most of the time; OR: odds ratio; * Odds ratio significant, *p* < 0.05; ** Odds ratio significant, *p* < 0.01; *** Odds ratio significant, *p* < 0.0001; N-values reflect the total number of children, n represents the number of children in the risk group. ^b^ Children 3–<6-years: Estimate the risk of head of household, mother’s highest level of education, and province for %E from protein<AMDR, adjusting for gender. ^c^ Children 6–<10-years: Estimate the risk of the province and mother’s BMI for %E from protein<AMDR, adjusting for gender.

**Table 9:** Bivariate and multivariate logistic regression of sociodemographic factors on having a fat intake below and above the acceptable macronutrient distribution range for fat (AMDR) by age group.

	Bivariate Logistic Regression			Multivariate Logistic Regression (Adjusted for Gender and Ethnicity)		
	Children 1–<3-Yrs %E from Fat < AMDR OR (95% CI) N = 333 (n = 178)	Children 3–<6-Yrs %E from Fat > AMDR N = 514 (n = 98) OR (95% CI)	Children 6–<10-Yrs %E from Fat > AMDR N = 479 (n = 159) OR (95% CI)	Children 1–<3- Yrs %E from Fat < AMDR OR (95% CI) N = 333 (n = 178) No Results ^b^	Children 3–<6-Yrs %E from Fat > AMDR N = 514 (n = 98) OR (95% CI) ^c^	Children 6–<10- Yrs %E from Fat > AMDR N = 479 (n = 159) OR (95% CI) ^d^
Primary Caregiver ^a^						
Mother	Ref	Ref	Ref			
Grandparent	1.85 (0.80–4.29)	1.13 (0.59–2.15)	1.10 (0.50–2.40)			
Other (e.g., father, sibling, aunt)	2.40 (0.75–7.73)	0.58 (0.19–1.73)	0.72 (0.33–1.57)			
Gender						
Male	Ref	Ref	Ref		Ref	Ref
Female	0.75 (0.47–1.21)	1.29 (0.76–2.18)	1.07 (0.64–1.78)		1.15 (0.65–2.03)	1.10 (0.65–1.84)
Head of household						
Father	Ref	Ref	Ref			
Mother	0.45 (0.20–1.04)	1.41 (0.68–2.93)	0.92 (0.43–1.95)			
Grandparent	0.97 (0.56–1.67)	1.68 (0.81–3.49)	1.21 (0.66–2.23)			
Other (e.g., aunt, uncle)	1.17 (0.48–2.88)	1.78 (0.70–4.53)	1.03 (0.41–2.62)			
Marital status of mother						
Married	Ref	Ref	Ref			
Other (e.g., unmarried, divorced)	1.75 (0.88–3.47)	1.23 (0.65–2.35)	0.67 (0.39–1.13)			
Mother’s highest education						
Did not complete grade 12	Ref	Ref	Ref			
Completed grade 12	0.95 (0.50–1.80)	0.94 (0.45–1.94)	1.88 (0.94–3.78)			
Qualification after grade 12	1.25 (0.55–2.86)	0.98 (0.43–2.23)	1.54 (0.67–3.52)			
Father’s highest education						
Did not complete grade 12	Ref	Ref	Ref		Ref	
Completed grade 12	1.09 (0.55–2.17)	0.43 (0.21–0.86) *	1.00 (0.60–1.66)		0.43 (0.21–0.87) *	
Qualification after grade 12	0.47 (0.14–1.53)	1.19 (0.64–2.24)	2.02 (0.77–5.27)		0.95 (0.45–2.02)	
Mother’s employment status						
Not employed/do not know	Ref	Ref	Ref			
Employed	1.15 (0.53–2.52)	1.34 (0.81–2.22)	1.35 (0.82–2.22)			
Father’s employment status						
Not employed/do not know	Ref	Ref	Ref			
Employed	1.25 (0.66–2.36)	1.43 (0.81–2.54)	1.39 (0.91–2.13)			
Wealth Index Quintiles						
One/two/three	Ref	Ref	Ref		Ref	Ref
Four/five	1.06 (0.59–1.91)	1.87 (1.00–3.49) *	1.64 (1.02–2.62) *		1.71 (0.82–3.59)	1.36 (0.81–2.28)
Ethnicity						
Black African	Ref	Ref	Ref		Ref	Ref
Mixed ancestry	0.18 (0.10–0.33) ***	3.08 (1.69–5.62) **	1.91 (1.18–3.08) **		2.65 (1.38–5.09) **	1.96 (1.22–3.13) **
Province						
Western Cape	Ref	Ref	Ref		Co-linear with ethnicity	
Gauteng	2.65 (1.37–5.13) **	0.46 (0.24–0.86) *	0.77 (0.46–1.27)			
Type of residence						
Urban formal	Ref	Ref	Ref			Ref
Urban informal	1.43 (0.81–2.54)	0.52 (0.26–1.04)	0.32 (0.16–0.65) **			0.44 (0.20–0.96) *
Rural	0.93 (0.50–1.74)	1.33 (0.74–2.38)	0.82 (0.46–1.44)			0.65 (0.38–1.12)
Mother’s BMI						
Underweight/normal weight	Ref	Ref	Ref			
Overweight	0.99 (0.55–1.79)	0.96 (0.47–1.97)	0.71 (0.37–1.38)			
Obese	0.76 (0.43–1.34)	0.98 (0.49–2.00)	1.29 (0.79–2.10)			
Hunger scale						
Total score = 0: No risk	Ref	Ref	Ref			Ref
1–4: At risk of hunger	1.46 (0.80–2.68)	1.06 (0.56–2.02)	0.59 (0.29–1.19)			0.58 (0.29–1.15)
5–8: Food shortage in house	1.32 (0.64–2.73)	0.56 (0.28–1.12)	0.50 (0.28–0.87)*			0.54 (0.30–0.97) *

^a^ Primary caregiver: person who looks after child most of the time; ^b^ No results since there was a strong relationship between province and ethnicity; OR: odds ratio; * Odds ratio significant, *p* < 0.05; ** Odds ratio significant, *p* < 0.01; *** Odds ratio significant, *p* < 0.0001; N-values reflect the total number of children, n represents the number of children in the risk group. ^c^ Children 3 –<6-years: Estimate the risk of father’s highest level of education and the risk according to the wealth index quintiles for %E from fat > AMDR, adjusting for ethnicity and gender. ^d^ Children 6–<10-years: Estimate the risk according to wealth index quintiles, the type of residence and the risk according to the hunger scale for %E from fat > AMDR, adjusting for ethnicity and gender.

**Table 10:** Bivariate and multivariate logistic regression of sociodemographic factors on having a free sugars intake above the recommended 10%E by age group.

	Bivariate Logistic Regression			Multivariate Logistic Regression (Adjusted for Gender and Ethnicity)		
	Children 1–<3-Yrs %E from Total Free Sugars >10%E N = 333 (n = 126) OR (95% CI)	Children 3–<6-Yrs %E from Total free Sugars >10%E N = 514 (n = 225) OR (95% CI)	Children 6–<10- Yrs %E from Total Free Sugars >10%E N = 479 (n = 214) OR (95% CI)	Children 1–<3-Yrs %E from Total Free Sugars >10%E N = 333 (n = 126) OR (95% CI) ^b^	Children 3–<6-Yrs %E from Total Free Sugars >10%E N = 514 (n = 225) OR (95% CI) ^c^	Children 6–<10- Yrs %E from Total Free Sugars >10%E N = 479 (n = 214) OR (95% CI) ^d^
Primary caregiver ^a^						
Mother	Ref	Ref	Ref			
Grandparent	1.16 (0.64–2.10)	0.97 (0.59–1.57)	1.03 (0.57–1.86)			
Other (e.g., father, sibling, aunt)	0.83 (0.28–2.44)	0.86 (0.41–1.82)	1.30 (0.68–1.50)			
Gender						
Male	Ref	Ref	Ref	Ref	Ref	Ref
Female	1.43 (0.80–2.57)	0.92 (0.58–1.47)	0.85 (0.53–1.38)	1.52 (0.77–3.00)	0.89 (0.57–1.40)	0.90 (0.54–1.51)
Head of household						
Father	Ref	Ref	Ref	Ref	Ref	
Mother	3.28 (1.28–8.45) *	1.35 (0.76–2.41)	0.67 (0.32–1.37)	3.31 (1.22–8.94) *	1.44 (0.75–2.73)	
Grandparent	2.89 (1.53–5.43) **	1.49 (0.95–2.34)	0.92 (0.58–1.45)	2.83 (1.51–5.32) **	1.45 (0.88–2.39)	
Other (e.g., aunt, uncle)	2.80 (0.98–8.00)	2.92 (1.22–7.02) *	0.76 (0.33–1.77)	2.70 (0.95–7.70)	2.79 (1.04–7.46) *	
Marital status of mother						
Married	Ref	Ref	Ref			
Other (e.g., unmarried, divorced)	1.74 (0.89–3.39)	1.04 (0.68–1.57)	0.74 (0.44–1.25)			
Mother’s highest education						
Did not complete grade 12	Ref	Ref	Ref			
Completed grade 12	1.40 (0.70–2.80)	1.11 (0.60–2.06)	1.03 (0.60–1.76)			
Qualification after grade 12	1.13 (0.47–2.71)	1.15 (0.65–2.03)	1.44 (0.65–3.18)			
Father’s highest education						
Did not complete grade 12	Ref	Ref	Ref			
Completed grade 12	1.42 (0.70–2.87)	1.06 (0.69–1.64)	1.36 (0.81–2.28)			
Qualification after grade 12	0.71 (0.27–1.88)	1.08 (0.62–1.88)	0.95 (0.43–2.08)			
Mother’s employment status						
Not employed/do not know	Ref	Ref	Ref		Ref	Ref
Employed	0.99 (0.47–2.08)	1.66 (1.02–2.70) *	2.09 (1.23–3.56) **		1.31 (0.80–2.14)	1.86 (1.08–3.22) *
Father’s employment status						
Not employed/do not know	Ref	Ref	Ref			Ref
Employed	0.84 (0.52–1.33)	0.70 (0.44–1.12)	1.60 (1.10–2.35) *			1.57 (1.02–2.42) *
Wealth index quintiles						
One/two/three	Ref	Ref	Ref			
Four/five	0.95 (0.51–1.76)	1.22 (0.73–2.02)	0.99 (0.63–1.57)			
Ethnicity						
Black African	Ref	Ref	Ref	Ref	Ref	Ref
Mixed ancestry	1.50 (0.80–2.81)	1.40 (0.77–2.55)	2.73 (1.64–4.56) **	1.21 (0.59–2.48)	1.28 (0.70–2.36)	2.59 (1.50–4.49) **
Province						
Western Cape	Ref	Ref	Ref			
Gauteng	0.99 (0.53–1.87)	0.82 (0.47–1.42)	0.56 (0.34–0.91) *			Co-linear with ethnicity
Type of residence						
Urban formal	Ref	Ref	Ref	Ref		
Urban informal	0.43 (0.23–0.81) *	1.01 (0.62–1.66)	0.67 (0.43–1.04)	0.50 (0.25–1.02)		
Rural	0.79 (0.42–1.47)	0.95 (0.62–1.45)	0.99 (0.65–1.49)	0.73 (0.36–1.45)		
Mother’s BMI						
Underweight/normal weight	Ref	Ref	Ref		Ref	
Overweight	1.33 (0.63–2.78)	0.42 (0.20–0.88) *	1.25 (0.71–2.22)		0.50 (0.23–1.11)	
Obese	1.00 (0.52–1.95)	0.87 (0.51–1.48)	1.38 (0.76–2.49)		1.04 (0.58–1.86)	
Hunger scale						
Total score = 0: No risk	Ref	Ref	Ref		Ref	
1–4: At risk of hunger	0.62 (0.30–1.29)	0.50 (0.29–0.84) **	0.64 (0.33–1.26)		0.47 (0.28–0.79) **	
5–8: Food shortage in house	0.82 (0.33–2.01)	0.45 (0.26–0.77) **	0.69 (0.39–1.19)		0.49 (0.27–0.86) *	

^a^ Primary caregiver: person who looks after child most of the time; OR: odds ratio; * Odds ratio significant, *p* < 0.05; ** Odds ratio significant, *p* < 0.01; *** Odds ratio significant, *p* < 0.0001; N-values reflect the total number of children, n represents the number of children in the risk group. ^b^ Children 1–<3-years: Estimate the risk of head of household and type of residence for %E from total free sugars > 10%E, adjusting for ethnicity and gender. ^c^ Children 3–<6-years: Estimate the risk of head of household, mother’s employment status, mother’s BMI and the risk according to the hunger scale for %E from total free sugars > 10%E, adjusting for ethnicity and gender. ^d^ Children 6–<10-years: Estimate the risk of the mother’s employment status as well as the father’s employment status for %E from total free sugars > 10%E, adjusting for ethnicity and gender.

## Supplementary Materials

The following are available online at https://www.mdpi.com/1660-4601/17/5/1717/s1, Table S1: Loading of household possessions included in the wealth index for 5 quintiles title. Table S2: Hunger scale items. Table S3: NCI method used in the study. Table S4: Sociodemographic and other characteristics of the 1–<10-year-old children for the different dietary recalls.

## References

[1] Nasreddine L.M., Kassis A.N., Ayoub J.J., Naja F.A., Hwalla N.C. (2018). Nutritional status and dietary intakes of children amid the nutrition transition: The case of the Eastern Mediterranean Region. Nutr. Res..

[2] Hwalla N., Al Dhaheri A.S., Radwan H., Alfawaz H.A., Fouda M.A., Al-Daghri N.M., Zaghloul S., Blumberg J.B. (2017). The Prevalence of Micronutrient Deficiencies and Inadequacies in the Middle East and Approaches to Interventions. Nutrients.

[3] Winichagoon P. (2015). Transition of maternal and child nutrition in Asia: Implications for public health. Curr. Opin. Clin. Nutr. Metab. Care.

[4] Conde W.L., Monteiro C.A. (2014). Nutrition transition and double burden of undernutrition and excess of weight in Brazil. Am. J. Clin. Nutr..

[5] Tzioumis E., Adair L.S. (2014). Childhood dual burden of under- and overnutrition in low- and middle-income countries: A critical review. Food Nutr. Bull..

[6] De Onis M., Branca F. (2016). Childhood stunting: A global perspective. Matern. Child Nutr..

[7] Mendez M.A., Adair L.S. (1999). Severity and timing of stunting in the first two years of life affect performance on cognitive tests in late childhood. J. Nutr..

[8] Singhal A. (2017). Long-Term Adverse Effects of Early Growth Acceleration or Catch-Up Growth. Ann. Nutr. Metab..

[9] Senekal M., Nel J.H., Malczyk S., Drummond L., Harbron J., Steyn N.P. (2019). Provincial Dietary Intake Study (PDIS): Prevalence and Sociodemographic Determinants of the Double Burden of Malnutrition in A Representative Sample of 1 to Under 10-Year-Old Children from Two Urbanized and Economically Active Provinces in South Africa. Int. J. Environ. Res. Public Health.

[10] Steyn N.P., McHiza Z.J. (2014). Obesity and the nutrition transition in Sub-Saharan Africa. Ann. N. Y. Acad. Sci..

[11] Popkin B.M. (2006). Global nutrition dynamics: The world is shifting rapidly toward a diet linked with noncommunicable diseases. Am. J. Clin. Nutr..

[12] Bishwajit G. (2015). Nutrition transition in South Asia: The emergence of non-communicable chronic diseases. F1000Res.

[13] Ochola S., Masibo P.K. (2014). Dietary intake of schoolchildren and adolescents in developing countries. Ann. Nutr. Metab..

[14] Bosu W.K. (2015). An overview of the nutrition transition in West Africa: Implications for non-communicable diseases. Proc. Nutr. Soc..

[15] Labadarios D., Steyn N.P., Maunder E., MacIntryre U., Gericke G., Swart R., Huskisson J., Dannhauser A., Vorster H.H., Nesmvuni A.E. (2005). The National Food Consumption Survey (NFCS): South Africa, 1999. Public Health Nutr..

[16] Temple N.J., Steyn N.P., Myburgh N.G., Nel J.H. (2006). Food items consumed by students attending schools in different socioeconomic areas in Cape Town, South Africa. Nutrition.

[17] MacKeown J.M., Pedro T.M., Norris S.A. (2007). Energy, macro- and micronutrient intake among a true longitudinal group of South African adolescents at two interceptions (2000 and 2003): The Birth-to-Twenty (Bt20) Study. Public Health Nutr..

[18] Feeley A., Pettifor J.M., Norris S.A. (2009). Fast-food consumption among 17-year-olds in the Birth to Twenty cohort. S. Afr. J. Clin. Nutr..

[19] Harris T., Malczyk S., Jaffer N., Steyn N. (2019). How well are adolescents in the Gouda District of the Western Cape meeting the South African food-based dietary guidelines for fat, sugar and sodium?. J. Consumer Sci..

[20] Statistics South Africa Mid-Year Population Estimates 2018. http://www.statssa.gov.za/?p=11341.

[21] Statistics South Africa Census 2011 Metadata. http://www.statssa.gov.za/census/census_2011/census_products/Census_2011_Metadata.pdf.

[22] ICF International (2012). Demographic and Health Survey Sampling and Household Listing Manual: MEASURE DHS.

[23] Steyn N.P., Labadarios D., Maunder E., Nel J., Lombard C., Directors of the National Food Consumption S. (2005). Secondary anthropometric data analysis of the National Food Consumption Survey in South Africa: The double burden. Nutrition.

[24] Filmer D., Pritchett L. (1998). Estimating wealth effects without expenditure data—Or tears: With an application to educational enrollments in the states of India. The World Bank Development Research Group.

[25] South African Medical Research Council (MRC) (2017). South Africa Demographic and Health Survey: 2016.

[26] Wehler C., Scott R., Anderson J. (1992). The community childhood hunger identification project: A model of domestic hunger-demonstration. J. Nutr. Educ..

[27] Burrows T.L., Martin R.J., Collins C.E. (2010). A systematic review of the validity of dietary assessment methods in children when compared with the method of doubly labeled water. J. Am. Diet Assoc..

[28] Tooze J.A., Kipnis V., Buckman D.W., Carroll R.J., Freedman L.S., Guenther P.M., Krebs-Smith S.M., Subar A.F., Dodd K.W. (2010). A mixed-effects model approach for estimating the distribution of usual intake of nutrients: The NCI method. Stat. Med..

[29] Herrick K.A., Rossen L.M., Parsons R., Dodd K.W. (2018). Estimating usual dietary intake from National Health and Nutrition Examination 5. Survey data using the National Cancer Institute method. National Center for Health Statistics. Vital. Health. Stat..

[30] Moshfegh A.J., Rhodes D.G., Baer D.J., Murayi T., Clemens J.C., Rumpler W.V., Paul D.R., Sebastian R.S., Kuczynski K.J., Ingwersen L.A. (2008). The US Department of Agriculture Automated Multiple-Pass Method reduces bias in the collection of energy intakes. Am. J. Clin. Nutr..

[31] Steyn N., Senekal M. (2004). The Dietary Assessment and Education Kit (DAEK) The Chronic Diseases of Lifestyle Unit of the South African Medical Research Council.

[32] Steyn N.P., Senekal M., Norris S.A., Whati L., Mackeown J.M., Nel J.H. (2006). How well do adolescents determine portion sizes of foods and beverages?. Asia Pac. J. Clin. Nutr..

[33] Neville M.C., Allen J.C., Archer P.C., Casey C.E., Seacat J., Keller R.P., Lutes V., Rasbach J., Neifert M. (1991). Studies in human lactation: Milk volume and nutrient composition during weaning and lactogenesis. Am. J. Clin. Nutr..

[34] Lee R.D., Nieman D.C. (2013). Nutritional Assessment.

[35] WHO Obesity: Preventing and Managaing the Global Epidemic (Report of a WHO Consultation). https://www.who.int/nutrition/publications/obesity/WHO_TRS_894/en/.

[36] Van Graan A.E., Chetty J.M., Links M.R. (2017). Food Composition Tables for South Africa.

[37] Institute of Medicine Dietary Reference Intakes: The Essential Guide to Nutrient Requirements. https://www.nap.edu/read/11537/chapter/1#iii.

[38] WHO Guideline: Saturated Fatty Acid and Trans-Fatty Acid Intake for Adults and Children. https://extranet.who.int/dataform/upload/surveys/666752/files/Draft%20WHO%20SFA-TFA%20guidelines_04052018%20Public%20Consultation(1).pdf.

[39] WHO Guideline: Sugars Intake for Adults and Children. https://www.who.int/nutrition/publications/guidelines/sugars_intake/en/.

[40] Kibblewhite R., Nettleton A., McLean R., Haszard J., Fleming E., Kruimer D., Te Morenga L. (2017). Estimating Free and Added Sugar Intakes in New Zealand. Nutrients.

[41] World Medical Association (2013). World Medical Association Declaration of Helsinki: Ethical principles for medical research involving human subjects. J. Am. Med. Assoc..

[42] Popkin B.M., Corvalan C., Grummer-Strawn L.M. (2020). Dynamics of the double burden of malnutrition and the changing nutrition reality. Lancet.

[43] Mameli C., Mazzantini S., Zuccotti G.V. (2016). Nutrition in the first 1000 days: The origin of childhood obesity. Int. J. Environ. Res. Public Health.

[44] Michaelsen K.F., Greer F.R. (2014). Protein needs early in life and long-term health. Am. J. Clin. Nutr..

[45] Larnkjaer A., Molgaard C., Michaelsen K.F. (2012). Early nutrition impact on the insulin-like grwoth factor axis and later health consequences. Curr. Opin. Clin. Nutr. Metab. Care.

[46] WHO Global Strategy for Infant and Young Child Feeding. https://www.who.int/nutrition/publications/infantfeeding/9241562218/en/.

[47] Lawrence R.A. (2011). Increasing breastfeeding duration: Changing the paradigm. Breastfeeding Med..

[48] Huh S.Y., Rifas-Shiman S.L., Taveras E.M., Oken E., Gillman M.W. (2011). Timing of solid food introduction and risk of obesity in preschool-aged children. Pediatrics.

[49] Chaponda A., Goon D.T., Hoque M.E. (2017). Infant feeding practices among HIV-positive mothers at Tembisa hospital, South Africa. Afr. J. Prim. Health Care Fam. Med..

[50] Kassier S., Veldman F. (2013). Cry, the beloved bottle: Infant-feeding knowledge and the practices of mothers and caregivers in an urban township outside Bloemfontein, Free State province. S. Afr. J. Clin. Nutr..

[51] Van Der Merwe S., Du Plessis L., Jooste H., Nel D. (2015). Comparison of infant-feeding practices in two health subdistricts with different baby-friendly status in Mpumalanga province. S. Afr. J. Clin..

[52] Smuts M., Wolmarans P. (2013). The importance of the quality of type of fat in the diet: A food-based dietary guideline for South Africa. S. Afr. J. Clin. Nutr..

[53] UNICEF, FFI (2014). Monitoring of flour fortification: The case of South Africa. http://www.ffinetwork.org/monitor/Documents/SouthAfricaCS.pdf.

[54] Labadarios D. (1999). The National Food Consumption Survey (NFCS): Children Aged 1–9 Years.

[55] Temple N., Steyn N.P. (2015). Community Nutrition Textbook for South Africa: A Rights-based Approach. Cape Town: Chronic Diseases of Lifestyle Unit.

[56] The Scientific Advisory Committee on Nutrition Recommendations on Carbohydrates, Including Sugars and Fibre, Published 17 July 2015. Public Health England. https://assets.publishing.service.gov.uk/government/uploads/system/uploads/attachment_data/file/445503/SACN_Carbohydrates_and_Health.pdf.

[57] Shroff M.R., Perng W., Baylin A., Mora-Plazas M., Marin C., Villamor E. (2014). Adherence to a snacking dietary pattern and soda intake are related to the development of adiposity: A prospective study in school-age children. Public Health Nutr..

[58] Bucher Della Torre S., Keller A., Laure Depeyre J., Kruseman M. (2016). Sugar-Sweetened Beverages and Obesity Risk in Children and Adolescents: A Systematic Analysis on How Methodological Quality May Influence Conclusions. J. Acad. Nutr. Diet..

[59] Hu F.B. (2013). Resolved: There is sufficient scientific evidence that decreasing sugar-sweetened beverage consumption will reduce the prevalence of obesity and obesity-related diseases. Obes. Rev..

[60] Orushka A. Sugar Sweeteened Beverages (SSB) Tax in South Africa: An Analysis of the Tax Design. http://researchspace.ukzn.ac.za/handle/10413/16470.

[61] Bourne L. (2007). South African paediatric food-based dietary guidelines. Matern. Child Nutr..

[62] Shisana O., Labadarios D., Rehle T., Simbayi L., Zuma K., Dhansay A., Reddy P., Parker W., Hoosain E., Naidoo P. (2014). South African National Health and Nutrition Examination Survey (SANHANES-1).

[63] South African Department of Health Government Gazette No.34029: Regulations Relating to Trans Fats in Foods. https://extranet.who.int/ncdccs/Data/ZAF_B17_Regulationtransfatfoodstuffs.pdf.

[64] Te Morenga L., Montez J.M. (2017). Health effects of saturated and trans-fatty acid intake in children and adolescents: Systematic review and meta-analysis. PLoS ONE.

[65] He F.J., MacGregor G.A. (2006). Harmful effects of salt in determining blood pressure: Meta-analysis of controlled trials. Hypertension.

[66] Hill J., Mchiza Z., Puoane T., Steyn N.P. (2018). Food sold by street-food vendors in Cape Town and surrounding areas: A focus on food and nutrition knowledge as well as practices related to food preparation of street-food vendors. J. Hunger Environ. Nutr..

[67] Pries A.M., Filteau S., Ferguson E.L. (2019). Snack food and beverage consumption and young child nutrition in low- and middle-income countries: A systematic review. Matern. Child. Nutr..

[68] Afshin A., Sur P.J., Fay K.A., Cornaby L., Ferrara G., Salama J.S., Mullany E.C., Abate K.H., Abbafati C., Abebe Z. (2019). Health effects of dietary risks in 195 countries, 1990–2017: A systematic analysis for the Global Burden of Disease Study 2017. Lancet.

