# Supplementary material for: Provincial Dietary Intake Study (PDIS): Energy and Macronutrient Intakes of Children in a Representative/Random Sample of 1–<10-Year-Old Children in Two Economically Active and Urbanized Provinces in South Africa
Source: Int J Environ Res Public Health. 2020 Mar 5;17(5):1717. doi: 10.3390/ijerph17051717 (PMC7084522; doi:10.3390/ijerph17051717)
Supplement: Supplementary file 1 [file ijerph-17-01717-s001.pdf]

The wealth index was constructed from 21 items that had a loading on the first three factors of greater than 0.3 or less than -0.3. The binary items with positive loadings on factor 1 are: have electricity, have an electric or gas stove, cook with electricity, have a working refrigerator, have a working TV, flush toilet in house, live in a brick house, tap water inside the house, tap water in yard, flush toilet in yard, own a car, own a washing machine, own a microwave, own a computer, own a watch, own a vacuum cleaner, own a bicycle and the number of rooms in the house as an ordinal variable. The following items have negative loading on factor 1: drinking water comes from a communal tap, cook with paraffin and finally, the structure of the house is informal. Households were classified according to the asset index into quintiles.

Filmer, D.; Pritchett, L. Estimating wealth effects without expenditure data—or tears: with an application to educational enrollments in the states of India. *Demography*, **2001**, *38*, 115–132.

Table S1. Loading of household possessions included in the wealth index for 5 quintiles.

Household Possessions (%)	Gauteng Wt% (95% CI) (N=733)	Western Cape Wt% (95% CI) (N=593)	ALL Wt% (95% CI) (N=1326)	Asset group 1 Wt% (20.0%)	Asset group 2 Wt% (20.0%)	Asset group 3 Wt% (19.5%)	Asset group 4 Wt% (20.5%)	Asset group 5 Wt% (20.0%)
Refrigerator	82.7 (78.4-86.9)	85.3 (79.2-91.3)	83.5 (80.1-86.9)	42.9###	75.5	99.2	100.0	100.0
Stove (any type)	92.8 (88.6-97.0)	95.0 (92.7-97.4)	93.5 (90.7-96.4)	69.1###	98.4	100.0	100.0	100.0
Microwave	54.3 (46.9-61.6)###	68.1 (59.8-76.3)	58.8 (53.3-64.4)	19.3###	48.2	39.0	87.0	99.3
Washing machine	41.3 (32.8-49.8)###	66.4 (57.5-75.4)	49.6 (43.3-56.0)	13.7###	39.8	28.5	68.1	97.0
Vacuum cleaner	5.2 (1.6-8.9)###	22.8 (15.7-29.8)	11.1 (7.8-14.4)	2.0###	6.3	8.9	11.3	26.8
Radio	60.9 (55.7-66.1)	56.5 (49.0-64.0)	59.4 (55.2-63.6)	43.9###	50.3	59.1	70.3	73.4
Television	88.4 (84.7-92.0)	91.3 (87.1-95.4)	89.3 (86.6-92.1)	55.3###	91.1	100.0	100.0	100.0
Computer	17.8 (12.2-23.4)###	29.6 (21.2-38.0)	21.7 (17.1-26.3)	5.9###	14.1	12.3	19.9	56.2
Landline telephone	0.5 (0.0-1.1)###	11.0 (5.1-16.9)	4.0 (2.1-5.9)	0.0###	4.6	0.6	2.6	12.0
Watch	45.7 (38.3-53.2)###	63.4 (55.2-71.5)	51.6 (46.0-57.2)	30.5###	41.5	42.0	56.3	87.4
Cell phone	97.0 (95.2-98.8)	95.5 (93.0-97.9)	96.4 (95.1-97.9)	89.6###	96.3	98.5	98.5	99.5
Bicycle	15.7 (11.8-19.6)###	32.0 (23.5-40.5)	21.1 (17.3-24.9)	6.6###	21.0	11.1	18.1	48.6
Motorcycle	3.6 (1.7-5.6)###	11.1 (7.0 – 15.1)	6.1 (4.3-7.9)	1.3###	6.6	3.5	5.0	14.1
Car	27.1 (20.8-33.5)###	40.1 (30.9-49.3)	31.4 (26.3-36.6)	9.2###	19.3	17.9	32.9	77.6

Have electricity	93.9 (89.7-98.0)###	99.2 (98.3-100.0)	95.7 (92.9-98.4)	78.1###	100.0	100.0	100.0	100.0
Main cooking fuel (%)	N=732	N=588	1320					
Electric	91.7 (87.5-96.0)###	84.0 (78.6-89.3)	89.2 (85.9-92.5)	65.0###	81.9	98.8	100.0	100.0
Gas	1.7 (0.5-2.9)	14.7 (9.5-19.8)	6.0 (4.1-7.9)	10.6	18.1	1.2	0.0	0.0
Paraffin	6.1 (2.5-9.8)	0.2 (0.0-0.3)	4.2 (1.8-6.5)	21.0	0.0	0.0	0.0	0.0
Wood/coal	0.4 (0.0-1.0)	1.2 (0.1-2.2)	0.7 (0.2-1.2)	3.4	0.0	0.0	0.0	0.0
Type of toilet (%)	N=733	N=591	1324					
Flush toilet in house	57.5 (45.5-69.5)###	80.2 (73.0-87.3)	65.0 (56.7-73.3)	23.3###	55.9	74.1	80.0	91.6
Pit latrine	13.7 (5.4-22.0)	0.5 (0.1-0.8)	9.3 (3.8-14.8)	30.0	10.7	3.3	1.5	1.2
Bucket	0.6 (0.0-1.3)	2.4 (0.6-4.1)	1.2 0.5-1.9)	4.8	0.7	0.6	0.0	0.0
Chemical toilet	4.3 (2.2-6.3)	1.3 (0.0-3.8)	3.3 (1.7-4.9)	13.5	2.3	0.0	0.0	0.7
No facility	0.6 (0.0-1.5)	0.1 (0.0-0.3)	0.5 (0.0-1.1)	1.0	0.8	0.5	0.0	0.0
Other (flush outside)	23.3 (13.0-33.7)	15.5 (8.7-22.4)	20.8 (13.6-27.9)	27.5	29.7	21.5	18.6	6.4
Type of drinking water (%)	N=733	N=593	1326					
Tap in house	45.3 (35.1-55.5)###	79.3 (73.5-85.1)	56.6 (49.5-63.6)	24.4###	48.8	61.3	72.3	75.9
Tap in yard	45.6 (36.3-54.9)	8.6 (4.3-12.8)	33.3 (27.0-39.6)	38.1	47.4	36.8	26.1	18.0
Communal tap	8.8 (2.9-14.7)	4.5 (1.0-8.1)	7.4 (3.3-11.4)	36.1	1.0	0.0	0.0	0.0
Bottled water	0.3 (0.0 – 0.8)	7.6 (2.8-12.3)	2.7 (1.2-4.3)	1.3	2.8	1.9	1.6	6.2
Other (e.g. river/dam)	-	0.1 (0.0-0.1)	0.02 (0.0-0.04)	0.1	0.0	0.0	0.0	0.0
Type of dwelling (%)	N=733	N=593	1326					
Brick house/flat	72.3 (63.0-81.7)###	79.7 (73.8-85.6)	74.8 (68.3-81.2)	24.7###	55.8	93.5	99.9	100.0
Informal structure (shack/tin)	26.8 (17.6-36.0)	10.9 (5.1-13.7)	21.5 (15.3-27.8)	69.0	36.7	1.9	0.0	0.0
Other (mud/wooden/other)	0.8 (0.0-1.8)	9.4 (6.4-15.5)	3.7 (2.1-5.3)	6.3	7.5	4.6	0.1	0.0
Mean number rooms in house (95% CI)	2.9 (2.6 – 3.1)	2.9 (2.6 – 3.3)	2.9 (2.7 – 3.1)	2.0 (1.8 - 2.2) [E]	2.4 (2.1 - 2.6) [D]	2.7 (2.4 – 3.0) [C]	3.2 (2.9 – 3.4) [B]	4.2 (3.9 – 4.5) [A]
Mean number sleeping in house (95% CI)	5.2 (4.9 – 5.5)&&&	5.8 (5.5 – 6.2)	5.4 (5.2 – 5.6)	4.8 (4.4-5.1) [C]	5.2 (4.8-5.7) [B][C]	5.3 (5.0 – 5.7) [B]	5.8 (5.5 – 6.4) [A][B]	5.9 (5.4 – 6.2) [A]
Mean wealth index (95% CI)	0.5 (-0.2 – 1.2)&&&	1.3 (0.9 – 1.7)	0.8 (0.3 – 1.2)	-4.3 (-5.9--2.8) [D]	0.6 (0.6 – 0.7) [C]	1.9 (1.9 – 1.9) [B]	2.5 (2.5 – 2.5) [A]	3.0 (3.0 – 3.0) [A]

###Significant relationship between the variable and the province / wealth index category, Chi-square p-value<0.0001. [A], [B], [C], [D], [E]: significant difference between the mean values for different wealth index groups, Bonferroni multiple comparison, p<0.05. &&&Significant difference between

two provinces, independent t-test, $p<0.0001$. N-values reflect actual number of cases, estimates are adjusted using relevant weighting.

Table S2. Hunger scale items.

	YES	NO
1. Does your household ever run out of money to buy food?		
1a Has it happened in the past 30 days?		
1b Has it happened 5 or more days in the past 30 days?		
2. Do you ever rely on a limited number of foods to feed your children because you are running out of money to buy food for a meal?		
2a Has it happened in the past 30 days?		
2b Has it happened 5 or more days in the past 30 days?		
3. Do you ever cut the size of meals or skip any because there is not enough food in the house?		
3a Has it happened in the past 30 days?		
3b. Has it happened 5 or more days in the past 30 days?		
4. Do you ever eat less than you should because there is not enough money for food?		
4a. Has it happened in the past 30 days?		
4b. Has it happened 5 or more days in the past 30 days?		
5. Do your children ever eat less than you feel they should because there is not enough money for food?		
5a. Has it happened in the past 30 days?		
5b. Has it happened 5 or more days in the past 30 days?		
6. Do your children ever say they are hungry because there is not enough food in the house?		
6a. Has it happened in the past 30 days?		
6b. 5 or more days in the past 30 days?		
7. Do you ever cut the size of your children's meals or do they ever skip meals because there is not enough money to buy food?		
7a. Has it happened in the past 30 days?		
7b. Has it happened 5 or more days in the past 30 days?		
8. Do any of your children ever go to bed hungry because there is not enough money to buy food?		
8a. Has it happened in the past 30 days?		
8b. Has it happened 5 or more days in the past 30 days?		

Community Childhood Hunger Identification Project (CCHIP) questionnaire

Wehler, C.; Scott, R.; Anderson, J. The community childhood hunger identification project: a model of domestic hunger-demonstration. *J. Nutr. Education* **1992**, *24*, 295-355.

Table S3. NCI method used in the study.

The National Cancer Institute (NCI) method [1] that was developed to distinguish within-person from between-person variation, account for extreme intakes, including zero intake, and allow for adjustment for covariates and association analyses. The NCI method is used to adjust the measurement of the observed single 24-hour dietary intake data using data from the PDIS study, to establish usual intake, and thereby improve the validity of the results. Two additional 24-hour dietary recalls were completed on a subsample of 148 (2nd recall) and 146 (3rd recall) children in the sample. The last five EAs in each province, mainly for logistical reasons, were visited three times a week apart for this purpose. Parents of children also needed to indicate whether the 24-hour recall was less, same or more than the child's usual intake. The data obtained from the three 24-hour recalls of the subsample were used to adjust the observed distributions of the single 24-hour recall completed by the larger sample for the effects of random within-person variation.
Using the NCI method, the available 3-day 24-hour recalls for the subgroup were used to estimate within-person variance and remove it from the first 24-hour recall. The Balanced Repeated Replication (BRR) method [2] was used to do variance estimation with a Fay coefficient

of 0.3. Two pseudo primary sampling units (PSU) were created per stratum by randomly selecting half of the PSU (or EA) in each stratum into one pseudo-PSU, and the rest in a second pseudo-PSU [2,3]]. Therefore 6 original strata were maintained with 12 pseudo-PSUs, two per stratum. Consequently, 8 BRR weights were created, taking the original sampling weights as well as the age and gender of each child in consideration

When estimating usual intakes, covariates adjusted for in this study included province, type of residential area (urban formal, urban informal or rural), gender of the child and whether the intake of the 24-hour recall was less, the same or more than usual. The three age groups, namely 1 - <3-years, 3 - <6-years and 6 - <10-years were treated as subgroup options within the macros. The NCI method calculations should be interpreted at population level, and usual intakes for individuals within the group are not produced. The website accessed is: https://prevention.cancer.gov/research-groups/biometry/measurement-error-impact/software-measurement-error,

and the software selected are for estimating usual intake distribution, specifically for single regularly-consumed nutrients, and the percentage of energy intake from selected macronutrients. The macros used are the boxcox_survey.macro.v1.2.sas; brr_pvalue_ci_macro_v1.1.sas; distrib_bivariate.macro_v1.2.sas; nlmixed_bivariate_macro_v1.2.sas; nlmixed_univariate_macro_v1.2.sas and percentiles_survey.macro.v1.1.sas.

The BRR weights were calculated using the technique described in Herrick et al. [4].

References

1. Tooze, J.A.; Kipnis, V.; Buckman, D.W.; Carroll, R.J.; Freedman, L.S.; Guenther, P.M.; Krebs-Smith, S.M.; Subar, A.F.; Dodd, K.W. A mixed-effects model approach for estimating the distribution of usual intake of nutrients: The NCI method. *Stat. Med.* **2010**, *29*, 2857–2868, doi:10.1002/sim.4063.
2. Korn, E.L.; Graubard, B.I. *Analysis of health surveys*. John Wiley and Sons: New York, USA,1999.
3. Judkins, D.R. Fay's method for variance estimation. *J. Off. Stat.* **1999,** 6, 223–239.
4. Herrick, K.A.; Rossen, L.M.; Parsons. R.; Dodd, K.W. Estimating usual dietary intake from National Health and Nutrition Examination 5. Survey data using the National Cancer Institute method. National Center for Health Statistics. *Vital. Health. Stat*. **2018**, *2*, 1–63
5. National Cancer Institute. Software for measurement error in Nutrition Research, US Department of Health and Human Services, National Institutes of Health, Available online: https://prevention.cancer.gov/research-groups/biometry/measurement-error-impact/software-measurement-error (accessed on 20 January 2020)

Table S4. Sociodemographic and other characteristics of the 1-<10-year old children for the different dietary recalls.

Variables	Recall 1 (N = 1326)	Recall 2 / 3 (N = 148)	Cochran-Mantel-Haenszel (CMH) test*
**Primary caregiver**			
Mother	70.4	69.9	0.482
Father	5.0	1.4	
Grandparent	18.1	20.0	
Other (e.g. sibling, aunt)	6.6	8,7	
**Age in years**			
1-<3-years	26.0	25.4	0.735
3-<6-years	35.3	39.0	
6-<10-years	38.7	35.6	
**Gender**			
Male	49.3	54.3	0.294

Female	50.7	45.7	
**Head of household**			
Father	39.7	37.4	0.574
Mother	14.8	18.8	
Grandmother	24.0	26.8	
Grandfather	12.5	11.5	
Other (e.g. aunt, uncle)	9.0	5.6	
**Marital status of mother**			
Unmarried	39.0	49.8	0.001**
Married	30.4	32.0	
Divorced/ widowed	4.0	3.8	
Living together	25.5	14.5	
Other	1.2	0.0	
**Mother's highest level of education**			
Did not complete grade 12	55.9	57.4	0.603
Completed grade 12	30.8	31.2	
Qualification after grade 12	13.3	11.4	
**Father's highest level of education**			
Did not complete grade 12	29.1	29.8	0.601
Completed grade 12	31.9	35.3	
Qualification after grade 12	12.3	9.3	
Do not know	26.7	25.7	
**Mother's employment status**			
Yes	27.7	20.9	0.250
No	69.8	78.3	
Don't know/ not applicable	2.5	0.8	
**Father's employment status (%)**			
Yes	65.0	62.6	0.444
No	21.1	20.7	
Don't know/ not applicable	13.9	16.7	
**Wealth index quintiles**			
One	20.0	18.6	0.724
Two	20.0	20.4	
Three	19.9	25.2	
Four	20.2	18.5	
Five	20.0	17.4	
**Ethnicity**			
Black African	74.5	89.0	0.0007**
Mixed ancestry	24.1	9.9	
Other	1.5	1.1	
**Province**			
Gauteng Province	66.8	62.2	0.303
Western Cape	33.2	37.8	
**Type of residence**			
Rural	3.8	3.0	0.434
Urban formal	88.2	90.7	
Urban informal	8.0	6.4	
**Mother's BMI [39]**			
Underweight/normal	32.0	33.2	0.939

BMI=<18.5 & 18.5-24.9kgm^2			
Overweight BMI= 25-29.9 kgm^2	25.4	23.6	
Obese BMI ≥ 30kgm^2	42.6	43.2	
**Hunger scale [25]**			
Total score=0: No risk	54.9	61.2	0.8813
1-4: At risk of hunger	24.4	12.9	
5-8: Food shortage in house	20.7	25.9	

**What the child ate and drank the previous day, was it …?**	First 24-hour recall (N=1326)	Second 24-hour recall (N=148)	Third 24-hour recall (N=146)	CMH Chi-Square
The same as usual	75.8	79.7	77.4	
More than usual	7.4	8.1	10.3	P=0.18
Less than usual	16.8	12.2	12.3	

*CMH test: The Cochran–Mantel–Haenszel test is used for repeated data. It will establish whether there is a consistent difference in the weighted proportions across the repeats (http://www.biostathandbook.com/cmh.html).
